# Autism-associated protein POGZ controls ESCs and ESC neural induction by association with esBAF

**DOI:** 10.1186/s13229-022-00502-9

**Published:** 2022-06-01

**Authors:** Xiaoyun Sun, Linxi Cheng, Yuhua Sun

**Affiliations:** 1 Institute of Hydrobiology, Chinese Academy of Sciences, Wuhan, 430070 China; 2 University of Chinese Academy of Sciences, Beijing, 100010 China; 3 Innovation Academy for Seed Design, Chinese Academy of Sciences, Beijing, China

## Abstract

**Background:**

The *POGZ* gene has been found frequently mutated in neurodevelopmental disorders (NDDs), particularly autism spectrum disorder (ASD) and intellectual disability (ID). However, little is known about its roles in embryonic stem cells (ESCs), neural development and diseases.

**Methods:**

We generated *Pogz−/−* ESCs and directed ESC differentiation toward a neural fate. We performed biochemistry, ChIP-seq, ATAC-seq, and bioinformatics analyses to understand the role of POGZ.

**Results:**

We show that POGZ is required for the maintenance of ESC identity and the up-regulation of neural genes during ESC differentiation toward a neural fate. Genome-wide binding analysis shows that POGZ is primarily localized to gene promoter and enhancer regions. POGZ functions as both a transcriptional activator and repressor, and its loss leads to deregulation of differentiation genes, including neural genes. POGZ physically associates with the SWI-SNF (esBAF) chromatin remodeler complex, and together they modulate enhancer activities via epigenetic modifications such as chromatin remodeling and histone modification. During ESC neural induction, POGZ-mediated recruitment of esBAF/BRG1 and H3K27ac are important for proper expression of neural progenitor genes.

**Limitations:**

The genotype and allele relevant to human neurodevelopmental disorders is heterozygous loss of function. This work is designed to study the effects of loss of POGZ function on ESCs and during ESC neural induction. Also, this work lacks of in vivo validation using animal models.

**Conclusions:**

The data suggest that POGZ is both a transcription factor and a genome regulator, and its loss leads to defects in neural induction and neurogenesis.

**Supplementary Information:**

The online version contains supplementary material available at 10.1186/s13229-022-00502-9.

## Background

Autism risk gene *POGZ* encodes a transcription factor that contains multiple domains, including a zinc-finger cluster, an HP1-binding motif, a centromere protein B-DB domain, and a transposase-derived DDE domain [1]. Biochemical studies have shown that POGZ may function as a transcriptional repressor as it is a reader of heterochromatin-associated mark H3K9me2/3 [2], is associated with heterochromatin proteins (HP1s) [1], and inhibits transcription by an in vitro luciferase assay [3]. POGZ may play important roles in chromatin regulation, mitotic progression, and DNA repair via HP1 proteins. Interestingly, *POGZ* is one of the most recurrently mutated genes in patients with neurodevelopmental disorders and neuropsychiatric disorders, including schizophrenia, neuroectodermal-derived intellectual disability, and ASD [3–13]. However, the underlying etiology of NDDs caused by *POGZ* mutation remains elusive.

ASD and ID are characterized by early life onset with aberrant brain function, suggesting that there are neural developmental defects in children patients. In mice, *Pogz* mRNA is abundantly expressed during early gestation and reaches its maximum expression on embryonic day (E) 9.5. Specifically, *Pogz* is broadly expressed in the developing cortex [14, 15]. *Pogz−/−* mice are early embryonic lethal, and *Pogz−/−* embryos are absorbed as early as E10.5 when neurogenesis begins. These observations suggest that POGZ may play an important role in embryonic neural induction and development.

The SWI/SNF ATP-dependent remodelers comprise two ATPase subunits SMARCA4/BRG1 and SMARCA2/BRM and core members such as SMARCC1/BAF155, ARID1A/BAF250a and SMARCD1/BAF60a [16]. The BAF complex is known to play key roles in proliferation and differentiation in a number of different tissues and cell types, including embryonic stem cells and neural stem cells [17, 18]. In mESCs, it is called “esBAF” whose role in the maintenance of ESCs has been firmly established [19–28]. The BAF complex is highly associated with neural developmental diseases, and GWAS analyses of ASD and schizophrenia patients have revealed mutations in BAF components such as BAF155 and BAF250 [17, 29].

In this work, we hypothesized that POGZ plays important roles in the maintenance of ESCs and during ESC neural induction. We generated *Pogz−/−* ESCs and performed biochemistry, bioinformatics, and functional analyses to understand the role of POGZ. We show that POGZ is both a transcription factor and an epigenome regulator. POGZ physically associates with the esBAF complex and recruits it to neural genes during ESC differentiation toward a neural fate. Loss of POGZ leads to change in chromatin state and enhancer activity, resulting in abnormal gene expression.

## Methods

### ES cell culture

Mouse embryonic stem cells (mESCs) R1 were maintained in Dulbecco’s Modified Eagle Medium (DMEM, BI, 01–052-1ACS) containing 20% knockout serum replacement (KSR, Gibco, 10,828,028), 1 mM sodium pyruvate (Sigma, S8636), 2 mM L-Glutamine (Sigma, G7513), 1,000 U/mL leukemia inhibitory factor (LIF, Millipore, ESG1107), and penicillin/streptomycin (Gibco, 15,140–122) at 37 °C with 5% CO2. Cells were routinely propagated by trypsinization and replated every 3–4 days, with a split ratio of 1:6.

### Embryoid body formation

ESCs differentiation into embryoid bodies (EBs) was performed in attachment or suspension culture in medium lacking LIF and knockout serum replacement (KSR), as described previously [30].

### Generation of Pogz−/− ESCs

*Pogz−/−* mESCs were generated by CRISPR/Cas9 technology. Briefly, we designed gRNAs on exon 2 of the *Pogz* gene by using the online Web site http://crispr.mit.edu/. The gRNA sequence is 5′-CGACCTGTTTATGGAATGTGAGG-3′. The guide sequence oligos were cloned into pSpCas9(BB)-2A-puro containing Cas9 and the sgRNA scaffold [31]. The plasmid without addition of the gRNA was used as control. The plasmids containing the sgRNA sequence or control plasmids were transfected into mESCs using Neon transfection system according to the manufacturer’s instructions. 48 h later, transfected mESCs were selected with 1 µg/mL puromycin for 4–5 days. Most of cells died within 4 days, and the remaining cells were allowed to proliferate. After 10–15 days selection, the single colony was picked up and passaged into 24-well plates.

DNA from single colonies from the passaged cells was extracted and used for genotyping by PCR. The PCR primer was designed to amplify 200–300 bp on either side of the sgRNA sites. The PCR products were ligated into pGEM-T vector and sequenced to determine the genotype of each single colony. We have successfully generated three mutant alleles (1 bp deletion, 7 bp insertion, and 284 bp insertion in exon 2 of the *Pogz* gene). Three homozygous *Pogz*−/− ESC lines were established: Mutation 1 (Mut1, 1 bp deletion), Mutation 2 (Mut2, 284 bp insertion), and Mutation 3 (Mut3, 7 bp insertion). One heterozygous ESC line (same allele as Mut1) was also included.

### Generation of 3 × Flag-POGZ restoring Pogz−/− mESC cell lines

The full-length *Pogz* cDNA (NM_172683.4) was amplified by PCR and then cloned into pCMV-3 × Flag vector. The full-length *Pogz* cDNA sequence containing N-terminal 3 × Flag sequence was subcloned into pCAG-IRES-Puro vector. To make stable transgenic cells, *Pogz−/−* mESCs were transfected with pCAG-IRES-Puro-3 × Flag-*Pogz* vector using Lipofectamine 2000 (Gibco, 11,668,019). 48 h later, cells were selected by 1 μg/mL puromycin. After 4–5 days drug selection, cells were expanded and passaged. Western blot assay was performed to confirm the transgenic cell line using FLAG antibodies.

### RNA preparation, RT-qPCR and RNA-seq

Total RNA from mESCs and ESC-derived EBs was extracted with a total RNA kit (Omega, R6834-01). A total of 1 μg RNA was reverse-transcribed into cDNA using the TransScript All-in-One First-Strand cDNA Synthesis SuperMix (TransGen Biotech, China, AT341). Quantitative real-time PCR (qRT-PCR) was performed using the TransStart® Tip Green qPCR SuperMix (TransGen Biotech, China, AQ-141). All experiments were repeated at least three times. The relative gene expression levels were calculated based on the 2^−∆∆Ct^ method. Data were shown as means ± S.D. The Student’s *t* test was used for the statistical analysis. The significance is indicated as follows: *, *p* < 0.05; **, *p* < 0.01; ***, *p* < 0.001.

For RNA-seq, control and Pogz mutant ESCs, and ESC-derived EBs were collected in tubes preloaded with Trizol (TransGen). RNAs were quantified by a NanoDrop instrument and were submitted to BGI Shenzhen (Wuhan, China) for mRNA enrichment, library construction, and sequencing (BGISeq 50SE). RNA-seq was performed in at least two technical repeats and two biological replicates, for control and mutant ESCs. *P* < 0.05 and a Log2 fold change > 1 was deemed to be differentially expressed genes (DEGs).

### Protein extraction and Western blot analysis

For protein extraction, ESCs or HEK293T cells were harvested and lysed in TEN buffer (50 mM Tris–HCl, 150 mM NaCl, 5 mM EDTA, 1% Triton X-100, 0.5% Na deoxycholate, supplement with Roche cOmplete Protease Inhibitor). The lysates were quantified by the Bradford method, and equal amount of proteins were loaded for Western blot assay. Antibodies used for WB were POGZ (Abcam, ab171934), anti-BRG1 (Proteintech, 21,634–1-AP), anti-HP1gamma (Proteintech, 11,650–2-AP), anti-CHD4 (Proteintech, 14,173–1-AP), anti-OCT4 (Proteintech, 60,242–1-Ig), anti-FLAG (F1804/F3165, Sigma, 1:1000), anti-MYC antibody (TransGen Biotech, HT101), and anti-HA (Abbkine, A02040, 1:1000). Briefly, the proteins were separated by 10% SDS-PAGE and transferred to a PVDF membrane. After blocking with 5% (*w*/*v*) non-fat milk for 1 h at room temperature, the membrane was incubated overnight at 4 °C with the primary antibodies. Then, the membranes were incubated with a HRP-conjugated goat anti-rabbit IgG (GtxRb-003-DHRPX, ImmunoReagents, 1:5000) and a HRP-linked anti-mouse IgG (7076S, Cell Signaling Technology, 1:5000) for 1 h at room temperature. The GE ImageQuant LAS4000 mini luminescent image analyzer was used for photographing. Western blot experiments were repeated at least two times.

Quantification of Western blot bands was performed by ImageJ software, according to the Web site: https://imagej.nih.gov/ij/docs/guide/146-30.html. Briefly, the rectangle tool was selected and used to draw a box around the lane, making sure to include some of the empty gel between lanes and white space outside of the band. All lanes were selected one by one. Once all lanes are defined, go to Plot lanes to generate histograms of each lane. Then, the relative values were calculated by dividing each value by the control lane. The value of the control bands was set at 1.

### Co-immunoprecipitation assay (co-IP)

Co-IPs were performed with the Dynabeads Protein G (Life Technologies, 10004D) according to the manufacturer’s instructions. Briefly, 1.5 mg Dynabeads was conjugated with antibodies or IgG overnight at 4 °C. Antibodies were used are: 10 μg IgG (Proteintech, B900610), or 10 μg anti-POGZ antibody, or 10 μg anti-FLAG antibody (Sigma, F3165/F1804), or 10 μg anti-HA antibody (Abbkine, A02040) or 10 μg anti-MYC antibody (TransGen Biotech, HT101). The next day, total cell lysates and the antibody-conjugated Dynabeads were incubated overnight at 4 °C with shaking. After three times of washing with PBS containing 0.1% Tween, the beads were boiled at 95 °C for 5 min with the 6 × protein loading buffer and the supernatant was collected for future WB analysis.

### Immunofluorescence assay

ESCs or ESC-derived cells were collected and fixed with 4% paraformaldehyde for half an hour at room temperature. Then, the cells were washed with PBST (phosphate-buffered saline, 0.1% Triton X-100) for three times, each for 15 min. Following the incubation with blocking buffer (5% normal horse serum, 0.1% Triton X-100, in PBS) for 2 h at room temperature, the cells were incubated with primary antibodies at 4 °C overnight. Antibodies used were POGZ (Abcam, ab171934), OCT4 (Proteintech, 60,242–1-Ig), NANOG (Bethyl, A300-397A), GATA6 (Abcam, ab175349), HP1 (Abcam, ab213167), and PH3 (CST, #9701). After three times of wash with PBST, the cells were incubated with secondary antibodies (1:500 dilution in blocking buffer, Alexa Fluor 488, Life Technologies) at room temperature for 1 h in the dark. The nuclei were counterstained with DAPI (Sigma, D9542, 1:1000). After washing with PBS for twice, the slides were mounted with 100% glycerol on histological slides. Images were taken by a Leica SP8 laser scanning confocal microscope (Wetzlar, Germany). About 10 images were taken for each slide. Quantification of IF staining was performed using ImageJ [30].

### Immunoprecipitation in combination with mass spectrometry

After Co-IP, the IP samples (immunoprecipitated by IgG or POGZ antibody or FLAG antibody) were run on SDS-PAGE gels and stained with the Coomassie Blue. Then, the entire lanes for each IP samples were cut off and transferred into a 15-mL tube containing deionized water. The mass spectrometry was done by Genecreate Biological Engineering Company (Wuhan, China). Briefly, the samples were digested into peptides by trypsin treatment overnight, followed by C18 column for desalination. Next, the peptides were dissolved and subjected to mass spectrometry analysis by AB SCIEX Triple TOF™ 5600 plus (USA), followed by identification ProteinPilot database search engine. With confidence level ≥ 95%, unique peptides ≥ 1, an average of 260 proteins were identified.

### Chromatin Immunoprecipitation (ChIP) and ChIP-seq

ChIP experiments were performed according to the Agilent Mammalian ChIP-on-chip manual as described [32]. Briefly, 1 × 10^8^ ES cells were fixed with 1% formaldehyde for 10 min at room temperature. Then, the reactions were stopped by 0.125 M glycine for 5 min with rotating. The fixed chromatin was sonicated to an average of 200–500 bp (for ChIP-Seq) or 500–1000 bp (for ChIP-qPCR) using the S2 Covaris Sonication System (USA). For ChIP-seq, chromatin was sheared for 15 min with peak power 135; duty factor 5.0; and cycles/burst 200. Then, Triton X-100 was added to the sheared chromatin solutions to a final concentration of 0.1%. After centrifugation, 50 μL of supernatants was saved as input. The remainder of the chromatin solution was incubated with Dynabeads previously coupled with 10 μg ChIP grade antibodies (POGZ, Abcam, Ab171934; FLAG, Sigma, F1804; H3K4me3, Abcam, ab1012; H3K27ac, Millipore, MABE647) overnight at 4℃ with rotation. Next day, after seven times of washing with the wash buffer, the complexes were reverse-cross-linked overnight at 65 ℃. DNAs were extracted by hydroxybenzene–chloroform–isoamyl alcohol and purified by a Phase Lock Gel (Tiangen, China). For ChIP-seq, the ChIPed DNA was dissolved in 15 μL distilled water. Library constructions and deep sequencing were done by the BGI Shenzhen (Wuhan, China). All ChIP-seq experiments were repeated at least two times using control and mutant ESCs.

For ChIP-PCR, the ChIPed DNA was dissolved in 100 μL distilled water. Quantitative real-time PCR (qRT-PCR) was performed using a Bio-Rad qPCR instrument. The enrichment was calculated relative to the amount of input. All experiments were repeated at least three times. The relative gene expression levels were calculated based on the 2^−∆∆Ct^ method. Data were shown as means ± S.D. The Student’s t test was used for the statistical analysis. The significance is indicated as follows: *, *p* < 0.05; **, *p* < 0.01; ***, *p* < 0.001.

### CUT and tag

The cleavage under targets and tagmentation (CUT&Tag) experiments were performed using the 3 × Flag-POGZ restoring *Pogz−/−* mESCs by the Frasergen Company (Wuhan, China) [33]. Aliquots of cells (500,000 cells) were washed twice in 1.5 mL wash buffer (20 mM HEPES pH 7.5; 150 mM NaCl; 0.5 mM spermidine; 1 × protease inhibitor cocktail) by gentle pipetting. Concanavalin A-coated magnetic beads (Bangs Laboratories) were prepared, and 10 µL of activated beads was added per sample and incubated at RT for 15 min. The supernatant was removed, and bead-bound cells were resuspended in 50–100 µL Dig-Wash buffer (20 mM HEPES pH 7.5; 150 mM NaCl; 0.5 mM spermidine; 1 × protease inhibitor cocktail; 0.05% digitonin) containing 2 mM EDTA and 5 µg FLAG antibodies (Sigma, F1804), rotating overnight at 4 °C. Next day, the primary antibody was removed using the magnet stand. To increase the number of protein A binding sites for the bound antibody, the pig anti-rabbit IgG antibody (Novusbio, NBP1-73,654) was diluted 1:50 in 50–100 µL of Dig-Wash buffer and cells were incubated at RT for 30 min. Cells were washed using Dig-Wash buffer to remove unbound antibodies. A 1:200 dilution of pA-Tn5 adapter complex (~ 0.04 µM) was prepared in Dig-med Buffer (0.05% digitonin, 20 mM HEPES, pH 7.5, 300 mM NaCl, 0.5 mM spermidine, 1 × protease inhibitor cocktail). After removing the liquid on the magnet stand, 50–100 µL was added to the cells with gentle vortexing, which was incubated with pA-Tn5 at RT for 1 h. Next, the cells were resuspended in 50–100 µL tagmentation buffer (10 mM MgCl2 in Dig-med Buffer) and incubated at 37 °C for 1 h. To stop tagmentation, 2.25 µL of 0.5 M EDTA, 2.75 µL of 10% SDS, and 0.5 µL of 20 mg/mL proteinase K were added to 50 µL of sample, which was incubated at 55 °C for 30 min, and then at 70 °C for 20 min to inactivate proteinase K. To extract the DNA, 122 µL Ampure XP beads were added to each tube with vortexing, quickly spun, and held 5 min. Beads were washed twice in 1 mL 80% ethanol. After allowing to dry ~ 5 min, 30–40 µL of 10 mM Tris pH 8 was added. To amplify libraries, 21 µL DNA was mixed with 2 µL of a universal i5 and a uniquely barcoded i7 primer33, using a different barcode for each sample. A volume of 25 µL NEBNext HiFi 2 × PCR Master Mix was added and mixed. The sample was placed in a thermocycler with a heated lid using the following cycling conditions: 72 °C for 5 min (gap filling); 98 °C for 30 s; 14 cycles of 98 °C for 10 s; and 63 °C for 30 s; final extension at 72 °C for 1 min and hold at 8 °C. Post-PCR clean-up was performed by adding 1.1 × volume of Ampure XP beads (Beckman Counter), and libraries were incubated with beads for 15 min at RT, washed twice gently in 80% ethanol, and eluted in 30 µL 10 mM Tris pH 8.0.

### ATAC-seq experiments

50,000 control ESCs and *Pogz−/−* ESCs in LIF-FBS medium were used for ATAC-seq assay. The experiments were performed in two technical replicates and two biological replicates (− 1 and + 284 bp homozygous). Library preparation and ATAC-seq experiments were done by the BGI Shenzhen (Wuhan, China). Libraries were paired-end-sequenced (2 × 75 bp) using an Illumina NextSeq 500 device.

### Protein–protein interaction assay using a rabbit reticulocyte lysate system

Protein–protein interaction assay using a rabbit reticulocyte lysate system has been described previously [32]. Tagged-POGZ, Tagged-POGZ mutants, Tagged-BRG1, Tagged-BRG1 mutants, and Tagged-BAF15 were synthesized using the TNT coupled reticulocyte lysate system according to the manual (Promega, L5020, USA). Briefly, 1 μg of circular pCS2 version of plasmids was added directly to the TNT lysates and incubated for 1.5 h at 30 °C. 1 μL of the reaction products was subjected to WB assay to evaluate the synthesized protein. For protein–protein interaction assay, 5–10 μl of the synthesized HA or FLAG tagged proteins was mixed in a 1.5-ml tube loaded with the 300 μl TEN buffer, and the mixture was shaken for 30 min at room temperature. Next, IP or pull-down assay was performed using Dynabeads protein G coupled with anti-FLAG or anti-HA antibodies as described above.

### Apoptosis analysis by flow cytometry

Annexin V-EGFP/PI (Propidium Iodide) Apoptosis Detection Kit (Yeason, 40303ES20) was used to detect apoptotic cells according to the manual. The late apoptosis and necrotic cells will be AnnexinV^+^/PI^+^. Briefly, ESCs were treated with trypsin without EDTA and washed twice by DPBS. Then, cells were washed with 1 × Binding Buffer and incubated with Annexin V-EGFP for 5 min at room temperature in the dark, followed by treatment with PI Staining Solution. Next, apoptotic cells were detected by flow cytometry (BD, AccuriC6). The apoptosis analysis was supported by Wan Yan from the Analysis and Testing Center of Institute of Hydrobiology, Wuhan.

### Quantification and statistical analysis

Data are presented as mean values ± SD unless otherwise stated. Data were analyzed using Student’s *t* test analysis. Error bars represent s.e.m. Differences in means were statistically significant when *p* < 0.05. Significant levels are: *p < 0.05; **P < 0.01, and ***P < 0.001.

### Bioinformatics

#### RNA-seq analysis

The RNA-seq data were processed through standard experimental and analytical pipelines. Raw.fastq files were first assessed for quality using FastQC software. Technical sequences and sequencing adapters were trimmed using Trimmomatic tool. Each sample produced more than 20 M clean reads and were mapped to the mm10 reference genome using HISTA. Then, Cufflinks was used to generate Fragments Per Kilobase of transcript per Million mapped reads (FPKM) table. DESeq2 was performed to calculate differentially expressed genes with fold change > 2 and *P* < 0.05. Volcano plots were drew with EnhancedVolcano package or ggplot2 package.

#### ChIP-seq analysis

ChIP-seq raw data were filtered with SOAPnuke filter -l 15 -q 0.5 -n 0.01 -Q 2 -c 21. After filtering, the clean data were mapped to mm10 genome by SOAPaligner/SOAP2 (version: 2.21t) with Parameter: soap_mm_gz -p 4 -v 2 -s 35. Peaks were called with macs2 -g 2.73e9 -s 50 -p 1e-5 -m 10 30 -broad -B -trackline. MAnorm software was used for finding the differential peaks. The *P* value for each peak was calculated based on the Bayesian model, and the significant regions were picked up if |M|≧1 and *p* value≦10^−5^. The bedtools (version 2.30.0) was used for calculating the differential peaks from two MAnorm results. BigWig files were generated using deepTools (version 3.3.2). Heat maps were generated from the average of replicates using the deepTools. Peak centers were calculated based on the peak regions identified by MACS2.

To investigate the co-occupancy of POGZ and BRG1, we consulted previously published ChIP-seq data sets for BRG1 (GSE87820; GSM2808653) [24, 34]. To analyze POGZ at gene promoters and enhancers, we consulted previously published ChIP-seq data sets for H3K4me1 (GSM2575694), H3K27Ac (GSM2575695) [35]. KLF4 (GSM4072779), ESRRB (GSM4087822), OCT4 (GSM2341284), NANOG (GSM2521520), MED1 (GSM4060038), SS18 (GSM2521508), BAF250a (GSM3318684), and BAF155 (GSE69140). ChIP-seq data were also downloaded for this work.

### CUT-tag analysis

Technical sequences and sequencing adapters were trimmed using Trimmomatic tool. Raw data were evaluated using FastQC. Each sample was aligned in Bowtie2 with parameters -I 10 -X 1000 –dovetail –no-unal –very-sensitive-local –no-mixed –no-discordant. Non-aligning and multiple mappers were filtered out with Picard tools. Peaks were called using MACS2 (version 2.1.1) with the parameters -f BAMPE -B –call-summits –keep-dup all. The position of each peak in the peak total collection, the background value, and signal value (RPKM) of the peak were extracted for each sample, and the signal value was divided by the background value to obtain the enrichment fold of the peak. Then, the DESeq2 software was used to perform between-group different peak analysis, by setting the filtering threshold to: |log2FoldChange| ≧1, *P* value≦ 0.05. BigWig files were generated using deepTools (version 3.3.2). Heat maps were generated from averaged replicates using the deepTools.

### ATAC-seq analysis

ATAC-seq raw data were filtered out with SOAPnuke filter -l 5 -q 0.5 -n 0.1 -Q 2 -5 1 -c 25. The clean data were mapped to mm10 genome by Bowtie2 (version: 2.2.5) with parameters: bowtie2 -q –phred64 –sensitive –dpad 0 –gbar 99,999,999 –mp 1,1 –np 1 –score-min L,0,-0.1 -I 1 -X 1000 -p 16 -k 200. Peaks were calling with macs2 (version: 2.1.0) with peak calling parameter: macs2 -g mm -s 50 -B –trackline –nomodel –shift -100 –extsize 200. BigWig files were generated using the deepTools (version 3.3.2). The MAnorm software was used for finding differential peaks. The *P* value for each peak was calculated based on the Bayesian model, and the significant regions were picked up if ≧1 and *p*≦10^−5^. The bedtools (version 2.30.0) was used for calculating the differential peaks from two MAnorm results. BigWig files were generated using deepTools (version 3.3.2). Heat maps were generated from averaged replicates using the deepTools.

For comparison analysis of POGZ and BRG1, we downloaded the previously published ATAC-seq data sets for Brg1 KD ESCs (GSM1941485-6), Brg1 KO ESCs (GSM2341280), and OCT4 KO ESCs (GSE87819) [24, 35].

### Combined analysis of ATAC-seq and RNA-seq

BETA was used for the combined analysis of ATAC-seq and RNA-seq data [36].

## Results

### Loss of POGZ leads to compromised ESC self-renewal

**Fig. 1:**
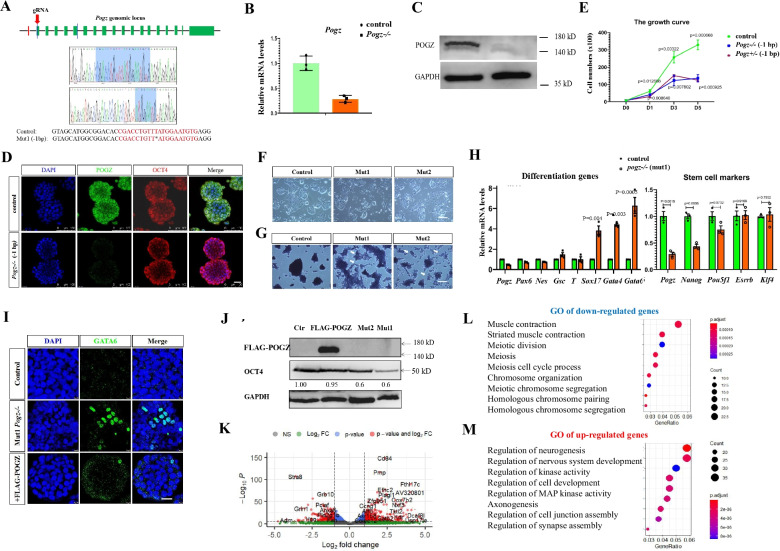
POGZ depletion leads to loss of ESC self-renewal. **A** (Up) Cartoon depicting the gRNA targeting sites at exon 2 of the mouse *Pogz* gene; (Bottom) Genotyping showing the mutant allele 1 (Mut1) of *Pogz* gene. **B** qRT-PCR showing the reduction of *Pogz* expression in mutant ESCs. **C** Western blot analysis of POGZ in control and *Pogz−/−* ESCs. **D** Double IF staining of OCT4 and POGZ showing that OCT4 levels were not significantly altered in early passage mutant ESCs. **E** The cell growth cure showing the proliferation defects of *Pogz−/−* and *Pogz+/− *ESCs compared to control ESCs. Mut1 ESCs at passage 12 were used for the assay.** F** Representative image showing morphology of control and late passage *Pogz−/−* ESCs (passage number is 18). White arrows pointing to the flattened differentiating cells. **G** Alkaline phosphatase staining of control and *Pogz−/−* ESCs. White arrows pointing to cells with reduced AP activities. **H** The mRNA expression levels of representative pluripotency-related, mesodermal, neuroectodermal, endodermal genes in control and *Pogz−/−* ESCs. **I** IF staining of GATA6 showing that GATA6 was abnormally expressed in Mut1 *Pogz*−/− ESCs. Restoring POGZ rescued the abnormal expression of Gata6. **J** WB showing that OCT4 levels were reduced in *Pogz−/−* ESCs, which can be reversed by reintroducing FLAG-POGZ. Bar: 25 μm. **K** Volcano plot showing the up- and down-regulated genes in the control and early passage *Pogz−/−* ESCs. The RNA-seq experiments were repeated two times. **L** GO analysis of up-regulated DEGs. **M** GO analysis of down-regulated DEGs. All WB and IF were repeated at least two times. qRT-PCR was repeated three times

To understand the function of POGZ, we generated *Pogz−/−* ESCs by CRISPR/Cas9 technology. The guide RNAs (gRNAs) were designed targeting the exon 2 of the *Pogz* gene (Fig. 1A). We have successfully generated three mutant alleles (1 bp deletion, 7 bp insertion, and 284 bp insertion in exon 2 of the *Pogz* gene) (Fig. 1A; Additional file 1: Figure S1A). ESCs with 1 bp deletion of *Pogz* gene were chosen to perform the majority of the subsequent experiments, and ESCs with other mutant alleles (+ 7/ + 284 bp designated as Mut2/3, respectively) were used to confirm the results when necessary. The qRT-PCR analysis showed that *Pogz* mRNA levels were greatly reduced in mutant ESCs (Fig. 1B). Western blotting results showed that POGZ protein was hardly detectable in mutant ESCs using POGZ antibodies of different resources (Fig. 1C), indicating that *Pogz* mutant alleles were functional null.

Early passage *Pogz−/−* ESCs (at passages 7–10) in the traditional self-renewal medium containing the LIF/KSR remained undifferentiated, as they displayed typical ESC-like domed morphology and abundantly expressed pluripotency marker OCT4 (Fig. 1D). However, late passage (> 15 passage) *Pogz−/−* and *Pogz* + */ − *ESCs displayed obvious cell growth and proliferation defects (Fig. 1E). Immunofluorescence (IF) staining of cell division marker phospho-histone H3 (PH3) showed that POGZ depletion decreased the number of PH3 positive cells (Additional file 1: Figure S1B–C). Apoptosis analysis by flow cytometry revealed that POGZ depletion leads to an increase in cell death (Additional file 1: Figure S1D). These observations were in line with that POGZ is essential for normal mitotic progression of cells [1], but additionally suggested that POGZ is required for cell survival or genomic stability. In our hand, *Pogz−/−* ESCs could be maintained and expanded for up to 20 passages, after which cells appeared difficult to proliferate. Meanwhile, a subset of late passaging *Pogz−/−* ESCs adopted a flatten morphology and lacked of tight cell contacts, indicating of spontaneous differentiation (Fig. 1F). This was confirmed by the reduced alkaline phosphatase AP activities (Fig. 1G), the decreased expression of pluripotency genes such as *Pou5f1* and *Nanog*, and the increased expression of differentiation genes such as *Sox17* and *Gata6* (Fig. 1H–J; Additional file 1: Figure S1E–F). Importantly, restoring FLAG-POGZ in *Pogz−/−* ESCs rescued the expression of GATA6 and OCT4 as well as the proliferation defects (Fig. 1I–J; Additional file 1: Figure S1E–F), demonstrating the specificity of the observed phenotypes.

To gain a global view of how POGZ is involved in the regulation of ESCs, we performed RNA-seq analysis for control and early passage *Pogz−/−* ESCs (at passage 10). A total of 1089 differentially expressed genes (DEGs) (fold change > 2, *p* < 0.05) were identified (Fig. 1K). Of which, 688 genes were up-regulated and 401 genes were down-regulated. Endodermal genes such as *Gata4/6*, *Sox17*, *Foxa2*, *Cited1*, *Cubn*, and genes implicated in neural differentiation and axonogenesis such as *App*, *En2*, *Insm1*, *Lama1*, *Meis1/2*, *Neurog3*, *Nefh*, *Nefl*, *Nefm*, *Ngfr*, *Nkx2.9*, *Nkx6.1*, *Nrg1*, *Nptx1*, *Robo1*, *Sox1*, *Zeb1*, and *Tcf12*, were up-regulated. Also, *Pogz−/−* ESCs showed increased expression of genes associated with cellular senescence and apoptosis, including *Cdkn2a* (INK4) and *Cdkn1a* (Cip1), and P53 target genes such as *Mdm2*, *Trp53inp1*, *Polk*, and *Ccng1*. Moreover, meiosis-specific homologous recombination genes such as *Smc1b*, *Dmc1*, and *Sycp3* were deregulated in *Pogz−/−* ESCs. Consistently, gene ontology (GO) analysis of up-regulated DEGs showed the enriched terms such as regulation of neurogenesis and nervous system development, axonogenesis, and synapse assembly, and GO of down-regulated DEGs revealed the enriched terms such as meiotic chromosome segregation and cell cycle (Fig. 1L-M).

### POGZ promotes neural progenitor genes during ESC neural induction

**Fig. 2:**
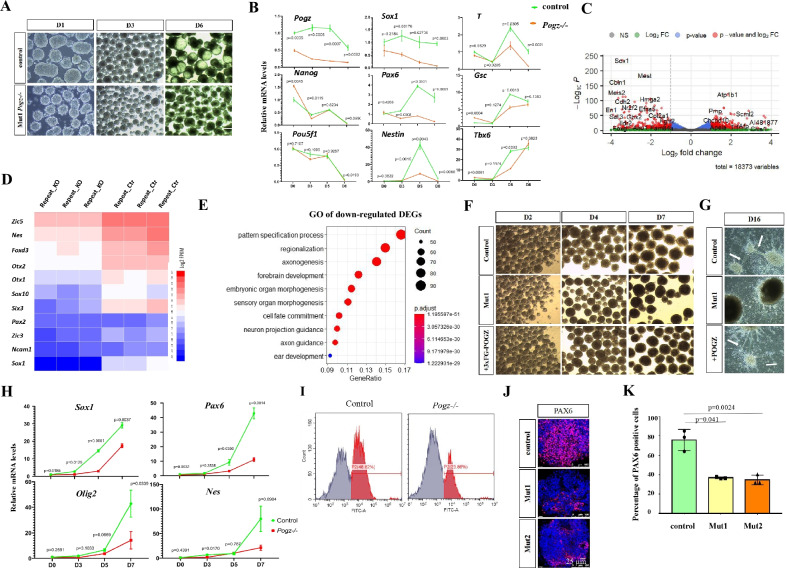
POGZ promotes ESC neural induction. **A** Morphology of day 1, 3, and 6 EBs from control and early passage Mut1 ESCs. **B** Time course qRT-PCR analysis of indicated genes. **C** Volcano plot showing the up- and down-regulated genes (fold change > 2) of day 6 EBs from control and *Pogz−/−* ESCs. **D** Heat map showing the expression of indicated neural genes. Three replicates are shown: KO for *Pogz−/−* ESCs, and Ctr for control ESCs. **E** GO analysis of DEGs of day 6 EBs from control and *Pogz−/−* ESCs. **F** Morphology of day 2, 4, 7 neurospheres from control-, *Pogz−/−*, and POGZ-restoring ESCs. **G** Morphology of day 16 neuronal cells from control-, *Pogz−/−*, and POGZ-restoring ESCs. White arrows pointing to the neuronal fibers. **H** Time course qRT-PCR analysis of indicated neural genes during control and Mut1 ESC directional differentiation toward neural progenitors. **I** Flow showing the percentage of PAX6-positive cells in control and *Pogz−/−* ESC-derived neurospheres. **J** IF results showing PAX6-positive cells in day 7 neural progenitors from control, Mut1, and Mut2 ESCs. Bar: 25 μm. **K** Quantitation of (J) by Image J. Flow and IF experiments were repeated at least two times. qRT-PCR was repeated at least three times

We went on to ask whether loss of POGZ leads to ESC pluripotency defects. To this end, we performed embryoid bodies (EBs) formation assay for control and early passage mutant ESCs. EBs from control ESCs were round and large, whereas *Pogz−/−* ESC-derived EBs were irregular in shape and smaller in size, suggesting that there was a defective formation of EBs (Fig. 2A; Additional file 1: Figure S2A). The qRT-PCR analysis was performed to examine the expression of germ layer genes for a time course of 8 days. Compared to controls, during *Pogz−/−* ESC EB formation, neural progenitor genes such as *Sox1*, *Nes,* and *Pax6* were significantly down-regulated (Fig. 2B).

To comprehensively understand how ESC pluripotency was affected in the absence of POGZ, we performed RNA-seq analysis for day 6 control and mutant ESC-derived EBs. A total of 1,230 DEGs were identified (Fig. 2C). Of which, 570 genes were down-regulated and 660 genes were up-regulated. Pluripotency genes such as *Pou5f1* and *Nanog*, neural genes such as *Pax6*, *Nes*, *Sox1*, *Foxd*3, and *Nrg1,* and early mesodermal genes such as *T, Tbx6*, and *Gsc* were down-regulated (Fig. 2D). By contrast, cardiac progenitor markers *Gata4*, *Nkx2.5*, *Foxf1*, *Tbx2/3*, *Tbx20*, and *Isl1* were up-regulated. Consistently, GO analysis of down-regulated DEGs showed the enriched terms such forebrain development, axonogenesis, and neuron projection guidance (Fig. 2E). KEGG analysis of down-regulated DEGs revealed the enriched terms such as axon guidance, Wnt signaling pathway, and pathways regulating pluripotency of stem cells (Additional file 1: Figure S2B).

The down-regulation of neural lineage genes was of great interest to us, provided that mutations of *POGZ* are known frequently linked with neurodevelopmental disorders [1, 7]. We hypothesized that POGZ is required for proper ESC neural induction by promoting neural progenitor genes (NPGs). To investigate this, we directly differentiated control and *Pogz−/−* ESCs into neurospheres for a time course of 7 days, using our previously published method [30]. The neurospheres from mutant ESCs appeared smaller than the counterparts from control ESCs (Fig. 2F). At day 16, the neuronal fiber-like structures were scarce in mutant ESC-derived neurons, whereas these fibers were abundant in controls (Fig. 2G; Additional file 1: Figure S2C). As shown in Fig. 2H, the expression levels of NPGs such as *Pax6* and *Nes* were markedly lower in *Pogz−/−* ESC-derived neural progenitors. Flow cytometry analysis showed that the percentage of Pax6 + cells in mutant neurospheres was only 1/2 that of control neurospheres (Fig. 2I). This was confirmed by IF staining using PAX6 antibodies (Fig. 2J–K).

Based on the above results, we concluded that POGZ is required for up-regulation of neural progenitor genes during ESC neural induction.

### POGZ physically associates with the esBAF complex

**Fig. 3:**
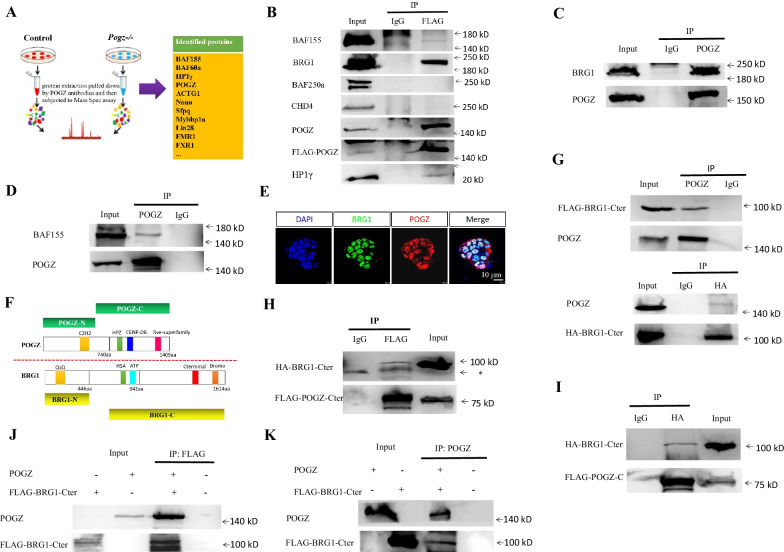
POGZ associates with esBAF. **A** Left: Cartoon showing the IP followed Mass Spec experiment. Right: representative POGZ interacting proteins identified. IP-Mass Spec experiments were repeated three times. **B** Co-IP results for FLAG-POGZ and the indicated proteins using FLAG-POGZ *Pogz−/−* ESCs. **C** Co-IP results confirming the interaction of endogenous POGZ and BRG1 in WT ESCs. **D** Co-IP results confirming the interaction of endogenous POGZ and BAF155 in WT ESCs. **E** Double IF staining of BRG1 and POGZ in WT ESCs. **F** Diagram showing the various truncated forms of BRG1 and POGZ used for mapping experiments. **G** Co-IP results showing that POGZ interacts with the C-terminal fragment of BRG1in 293 T cells. Up: IP POGZ; WB FLAG; Bottom: IP HA; WB POGZ. **H** POGZ-Cter pulled down the C-terminal fragment of BRG1 in 293 T cells. *: non-specific band. **I** HA-BRG1-Cter pulled down FLAG-POGZ-Cter in 293 T cells. **J-K** Co-IP results of in vitro synthesized POGZ and FLAG-BRG1-Cter. POGZ and FLAG-BRG1-Cter proteins were synthesized using a rabbit reticulocyte lysate system and subjected to pull-down analyses. Panel J: IP FLAG WB: POGZ; and panel K: IP POGZ WB: FLAG. All experiments were repeated at least two times, and shown are representative images

To understand the mechanisms by which POGZ may regulate ESCs, we performed IP combined with mass spectrometry assay (IP-Mass Spec) to identify its potential interacting proteins. A total of 85 proteins, including the known POGZ interacting protein HP1γ/CBX3, were identified by the IP-Mass Spec [1, 2]. Interestingly, members of the esBAF complex and its associated factors, such as SMARCC1/BAF155, SMARCD1/BAF60a, Nono, Sfpq, Mybbp1a, Actin, and ACTG1, were highly represented (Fig. 3A). BAF155, BAF60a, and ACTG1 are well-known members of esBAF chromatin remodeling complex and are involved in the regulation of ESC maintenance and differentiation [18, 19, 22, 37]. Nono, Sfpq, and Mybbp1a are previously known esBAF-associated proteins in ESCs [37]. Thus, our IP-Mass Spec experiments suggested that POGZ is closely related to the esBAF complex.

The co-IP experiments were performed to confirm the IP-Mass Spec data, using FLAG-POGZ *Pogz−/−* ESCs. The results showed that FLAG-POGZ was able to pull down HP1γ, BRG1, and BAF155, but not BAF250a and the NuRD complex member CHD4 (Fig. 3B). Furthermore, endogenous POGZ interacts with BRG1 and BAF155 but not CHD4 and its known binding partner ADNP (Fig. 3C–D; Additional file 1: Figure S3B–D). Double IF staining of BRG1 and POGZ clearly showed that they were co-localized in the nuclei of ESCs (Fig. 3E). Based on the biochemistry and IF data, we concluded that POGZ is highly associated with the esBAF/BRG1 complex, but not the NuRD/CHD4 complex.

As BRG1 is the core ATPase subunit of esBAF, we consider BRG1 to be the representative of esBAF complex in our following experiments. We went on to map the interaction domains between POGZ with BRG1 (Fig. 3F). The mapping results showed that the C-terminal fragment but not the N-terminal fragment of BRG1 is responsible to interact with POGZ (Fig. 3G; Additional file 1: Figure S3E-H). Further mapping showed that POGZ-Cter but not POGZ-Nter mediates its interaction with the C-terminal fragment of BRG1 (Fig. 3H–I; Additional file 1: Figure S3E).

Next, we asked whether POGZ directly interacts with BRG1. To this end, we used the TnT in vitro translation system to synthesize POGZ and FLAG-BRG1-Cter. When the in vitro synthesized POGZ and FLAG-BRG1-Cter were mixed together, FLAG-BRG1-Cter and POGZ can pull down each other (Fig. 3J–K). POGZ can also weakly interact with FLAG-BAF155 (Additional file 1: Figure S3I). Taken together, we concluded that POGZ physically associates with the esBAF complex.

### POGZ directly activates and represses target genes

To understand the function of POGZ, we performed ChIP-seq experiments using different sources of commercial POGZ antibodies. Unfortunately, these commercial antibody (Abcam, ab171934) failed to work in ChIP-seq experiments; we therefore performed FLAG ChIP-seq experiments in FLAG-POGZ *Pogz−/−* ESCs. The CUT&Tag, a newly developed enzyme-tethering technique for genome wide study, was also utilized in parallel [33].

**Fig. 4:**
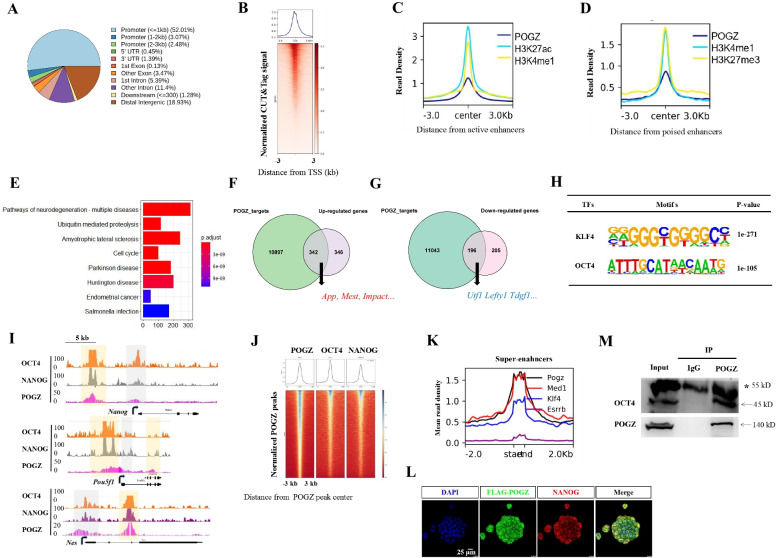
POGZ directly activates and represses target genes. **A** Pie chart showing the distribution of POGZ binding sites genome wide. **B** Heat map of POGZ CUT&Tag enrichment in a 6 kb window around the TSS. **C** Metaplot of POGZ, H3K4me1 and H3K27ac enrichment (normalized per million mapped reads) on ± 3 kb of genes that were bound by POGZ (27,343 peaks). **D** Metaplot of POGZ, H3K4me1 and H3K27me3 enrichment (normalized per million mapped reads) on ± 3 kb of genes that were bound by POGZ (1808 peaks). **E** KEGG analysis of POGZ-bound genes in ESCs. **F** Pie chart showing the overlap of POGZ targets and up-regulated genes. **G** Overlap of POGZ targets and down-regulated genes. **H** The enrichment of KLFs and OCT4/SOX2 binding motifs at POGZ-bound loci, as revealed by HOMER. **I** CUT&Tag and ChIP-seq snapshots showing the co-localization of POGZ, NANOG and OCT4 at the indicated loci. Gray: proximal TSS; Orange: distal regions. **J** Heat map of NANOG, OCT4 and POGZ signals across sites bound by POGZ (*n* = 11,000). Each row represents a 6 kb window centered on the peak midpoint. **K** Metaplot of POGZ, MED1, ESRRB and KLF4 enrichment (normalized per million mapped reads) on ± 2 kb window across all 231 super-enhancers that have been identified in ESCs by Whyte [41]. **L** Double IF staining of NANOG and FLAG-POGZ in ESCs. Bar: 25 μm. **M** IP results showing that OCT4 interacts with POGZ in ESCs. Star pointing to the IgG heavy chain. All IF and WB experiments were repeated at least two times

The peak intensity was much stronger by CUT&Tag than that of traditional ChIP-seq, showing that CUT&Tag outperformed the FLAG ChIP-seq. The snapshots of *Dcp1a* and *Chchd1* loci were shown for representatives (Additional file 1: Figure S4A). A total of 16,728 significantly enriched peaks were identified by the CUT&Tag. Analysis of CUT&Tag data revealed that POGZ was primarily localized to the proximal transcription start site (TSS) and distal regions (Fig. 4A–B). Further investigation showed that POGZ-occupied distal regions were enriched for poised and active enhancers (Fig. 4C–D; Additional file 1: Figure S4B).

A total of approximately 10,000 genes were bound by POGZ. KEGG analysis of POGZ targets revealed the enrichment of terms such as pathways of neurodegeneration, Parkinson and Huntington diseases (Fig. 4E). The KEGG results were in line with that *POGZ* mutations are frequently linked with neurodevelopmental or brain-related disorders.

The combined analysis of POGZ binding files and RNA-seq data allowed us to gain more insights into how POGZ regulates gene expression. Approximately 538 POGZ-bound genes (~ 5% of total targets) were deregulated in *Pogz−/−* ESCs. Of which, 342 genes were up-regulated and 196 were down-regulated, accounting for ~ 50% of all differentially up- and down-regulated genes (Fig. 4F–G). The up-regulated genes included *App*, *Mest*, and *Impact*, and the down-regulated ones included pluripotency-associated genes such as *Utf1* and *Lefty1*. These observations suggested that POGZ functions as both a transcriptional activator and repressor.

### POGZ function is associated with the maintenance of ESCs

POGZ is a typical transcription factor, suggesting that it can bind to DNA in a sequence-specific manner. Motif analysis of POGZ-bound sites by HOMER revealed a series of consensus DNA motifs of known TFs (Additional file 1: Figure S4C). The top ranked motifs were enriched for TFs such as SP1/2, KLFs, and OCT4 (Fig. 4H). KLF4 and OCT4 are well-known core pluripotency factors [38]. SP1, a previously identified POGZ interactor, is involved in transcription regulation [39].

As POGZ shares binding motifs with the core pluripotency factors, we asked whether POGZ is co-localized with NANOG/OCT4 genome wide. To this end, we consulted the published NANOG/OCT4 ChIP-seq data sets [24]. We found that there was an extensively overlap of POGZ and NANOG/OCT4 peaks genome-wide (Fig. 4I–J). Interestingly, broad POGZ peaks (3–5 kb) were observed when examining individual genes (Fig. 4I; Additional file 1: Figure S4B). This feature of binding was analogous to the previously described super-enhancers that are marked by mediators, ESRRB, KLF4, and P300/H3K27Ac [40, 41]. We thus speculated that POGZ is localized to super-enhancers. Our global meta-analysis showed that POGZ is indeed enriched at super-enhancers, similar to ESRRB, KLF4, and Med1 (Fig. 4K; Additional file 1: Figure S4D).

The co-localization of POGZ and OCT4/NANOG prompted us to ask whether they interact with each other in ESCs. Double IF staining showed that POGZ was clearly co-localized with NANOG in the nuclei of ESCs (Fig. 4L). The results of co-IP showed that POGZ could readily pull down endogenous OCT4 (Fig. 4M). The above results suggested that POGZ plays an important role in the maintenance of ESCs, which is in line with that loss of POGZ leads to compromised ESC stemness.

### POGZ recruits BRG1/esBAF to neural genes

**Fig. 5:**
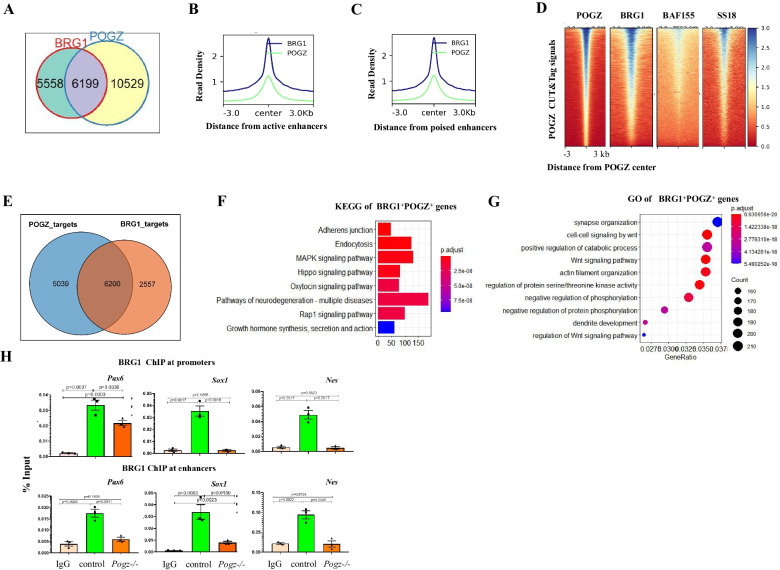
POGZ recruits esBAF/BRG1 to neural progenitor genes. **A** Pie chart showing the overlap of POGZ and BRG1 peaks. **B** Metaplot of POGZ and BRG1 enrichment (normalized per million mapped reads) across active enhancers (H3K4me1/H3K27ac). **C** Metaplot of POGZ and BRG1 enrichment (normalized per million mapped reads) across poised enhancers (H3K4me1/H3K27me3). A 6 kb window centered on the peak midpoint was shown. **D** Heat map view for distribution of POGZ, BRG1, BAF155 and SS18 signals in a ± 3 kb regions across sites bound by POGZ (*n* = 16,728). **E** Pie chart showing the overlap of POGZ and BRG1 target genes. **F** KEGG terms of POGZ^+^ BRG1^+^ genes. **G** GO terms of POGZ^+^ BRG1^+^ genes. **H** ChIP-PCR results showing BRG1 enrichment at promoter and enhancer regions of the indicated genes in day 5 neurospheres from control and *Pogz−/−* ESCs

As POGZ physically interacts with BRG1 in ESCs, we asked whether they were co-localized genome wide. POGZ and BRG1 peaks exhibited very similar distribution patterns (Additional file 1: Figure S5A). Analysis of CUT&Tag and ChIP-seq data showed that POGZ and BRG1 peaks were extensively overlapped (Fig. 5A). Approximately 37% (6199/16,728) of POGZ peaks were overlapped with 53% (6199/11,757) of BRG1 peaks. POGZ and BRG1 co-occupied sites were enriched at poised and active enhancers (Fig. 5B–C). BRG1 is the core enzymatic components of the esBAF, which included many other subunits such as BAF155, BAF60a, and SS18. Global meta-analysis showed that POGZ, BRG1, BAF155, and SS18 were co-localized genome wide (Fig. 5D; Additional file 1: Figure S5B).

We plotted POGZ CUT&Tag and BRG1 ChIP-seq reads in a ± 3 kb region surrounding the TSS and divided POGZ- and BRG1-bound genes into three categories (cluster A: POGZ^+^BRG1^+^; cluster B: POGZ^+^BRG1^−^; cluster C: POGZ^−^BRG1^+^) (Fig. 5E). GO analysis of POGZ^+^BRG1^+^ genes showed the enriched terms such as pathways of neurodegeneration and related diseases, and KEGG of these genes showed the enriched terms such as synapse organization, Wnt signaling, and dendrite development (Fig. 5F–G). Of note, GO analysis of POGZ^+^BRG1^−^ genes revealed the enriched terms such as double-strand DNA repair, rRNA and ncRNA processing. These results suggested that POGZ requires BRG1 to regulate neural-related process, while its ability to regulate RNA processing and genome stability does not require BRG1 (Additional file 1: Figure S5C–D).

It is known that BRG1 recruitment to neural genes by TFs is essential for proper neural development [29, 42]. We speculated that POGZ functions to recruit BRG1 to neural progenitor genes during ESC neural induction, and its loss leads to reduced BRG1 occupancy, resulting in failure to up-regulation of these neural genes. The ChIP-PCR analysis showed that BRG1 occupancy at promoters and enhancers of neural progenitor genes was markedly reduced in day 5 *Pogz−/−* ESC-derived neurospheres compared to the control counterparts (Fig. 5H; Additional file 1: Figure S5E). We concluded that POGZ-mediated BRG1 recruitment is important for ESC neural induction.

### POGZ promotes chromatin accessibility by association with BRG1 at enhancers

The esBAF is a well-known chromatin remodeler complex that regulates ESC chromatin state [22]. Considering the close relationship between POGZ and BRG1/esBAF, we asked whether loss of POGZ leads to change of chromatin accessibility, by performing ATAC-seq analysis for control and early passage *Pogz−/−* ESCs (passage number is 10).

**Fig. 6:**
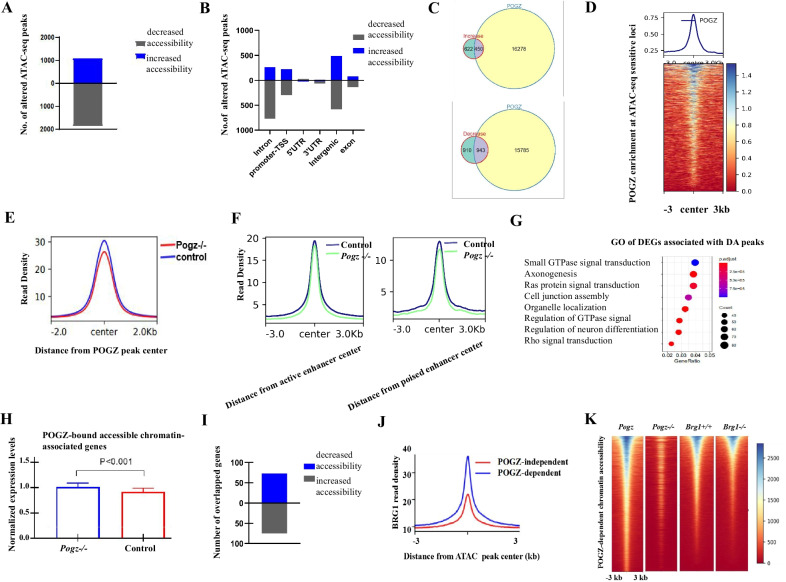
POGZ regulated-chromatin accessibility is linked with BRG1. **A** Bar chart showing number of altered ATAC-seq peaks. Blue color: ATAC-seq peaks showing increased signals in *Pogz−/−* ESCs; Black color: ATAC-seq peaks showing decreased signals in *Pogz−/−* ESCs. **B** Bar chart showing the genome-wide distribution feature of altered ATAC-seq peaks. **C** Up: Pie chart showing the overlap of POGZ peaks and ATAC-seq peaks showing increased signals in *Pogz−/−* ESCs; Bottom: Pie chart showing the overlap of POGZ peaks and ATAC-seq peaks showing decreased signals in *Pogz−/−* ESCs. **D** Heat map showing that POGZ signals were enriched at ATAC-seq hypersensitive sites. **E** Metaplot showing that chromatin accessibility is reduced at all POGZ-bound loci. **F** Metaplot showing that chromatin accessibility is moderately reduced at both active and poised enhancers, in the absence of POGZ. **G** GO analysis of DEGs associated with DA peaks. **H** Bar graph showing that POGZ-bound accessible chromatin-associated genes were slightly up-regulated in *Pogz−/−* ESCs. **I** Bar chart showing the overlap of DEGs and genes with altered chromatin accessibility. Seventy-three up-regulated genes were associated with DA regions showing increased chromatin accessibility, and 75 down-regulated genes were associated with DA regions showing decreased chromatin accessibility. **J** Metaplot showing that BRG1 is highly enriched at POGZ-dependent ATAC sites. **K** Heat map showing a reduction of ATAC-seq signals at POGZ-dependent ATAC sites in BRG1-depleted ESCs

Global meta-analysis showed that ATAC-seq signals were largely comparable between control and *Pogz−/−* ESCs (Additional file 1: Figure S6A). Nevertheless, a total of 2,930 differentially accessible (DA) peaks were identified. In the absence of POGZ, 1072 DA peaks showed increased chromatin accessibility and 1853 DA peaks showed decreased accessibility (Fig. 6A–B). Roughly half of the DA peaks were overlapped with sites bound by POGZ (Fig. 6C–D), and in the absence of POGZ, chromatin became less accessible at these loci (Fig. 6E; Additional file 1: Figure S6B). Bioinformatics analysis of POGZ-dependent DA peaks revealed that the top motifs were the putative ones bound by pluripotency factors SOX2, OCT4, and KLF4 (Additional file 1: Figure S6C).

Next, we defined putative enhancers in WT ESCs with H3K4me1 and H3K27ac and observed that there was a reduction of chromatin accessibility at both active and poised enhancers in mutant ESCs using a twofold change cutoff (Fig. 6F). By contrast, there was no change of chromatin accessibility around proximal TSS regions (Additional file 1: Figure S6D). This observation suggested that POGZ primarily promotes enhancer activities by rendering chromatin more accessible.

A total of 1962 genes associated with DA peaks were identified. GO analysis of the 1962 genes revealed the enriched terms such as axonogenesis and regulation of neuron differentiation (Fig. 6G). At a global level, POGZ-bound accessible chromatin-associated genes were slightly up-regulated in *Pogz−/−* ESCs (Fig. 6H). To further explore the relationship between chromatin accessibility and transcription, we performed a combined analysis of ATAC-seq and RNA-seq data, using BETA software [36]. A total of 148 overlapped genes were identified. Of which, 73 up-regulated genes were associated with DA regions showing increased chromatin accessibility and 75 down-regulated genes were associated with DA regions showing decreased chromatin accessibility (Fig. 6I). GO analysis of up-regulated genes revealed the enriched terms such as regulation of neuron differentiation and tissue development (Additional file 1: Figure S6E). As shown in Figure S6F, neural genes such as *Nrg1*, *Tcf4, Mest*, and *Slc6a1* displayed altered chromatin accessibility in the absence of POGZ.

Finally, we asked whether BRG1 is required for POGZ-regulated chromatin accessibility by examining the overlap of BRG1 ChIP-seq and POGZ-dependent ATAC-seq peaks. We found that BRG1 is highly enriched at POGZ-dependent ATAC-seq peaks (Fig. 6J). This observation suggested that POGZ may require BRG1 to shape chromatin accessibility at POGZ-bound sites. To further investigate this, we consulted the published ATAC-seq data for BRG1 [24]. When examining ATAC-seq signals at selected POGZ target sites in BRG1 knockout ESCs, we found a substantial reduction of chromatin accessibility (Additional file 1: Figure S6G). When extended to all POGZ-dependent ATAC sites, a reduction of ATAC-seq signals was observed in BRG1-depleted ESCs, similar to *Pogz−/−* ESCs (Fig. 6K). Thus, loss of BRG1 had a similar effect on chromatin accessibility to that by loss of POGZ, which suggested that POGZ promotes chromatin accessibility by association with BRG1.

### POGZ modulates enhancer activities in ESCs and during ESC neural induction

The above data indicated that POGZ is localized to gene promoter and enhancer regions where it regulates gene transcription. H3K4me3 and H3K27ac are well-known histone marks that are enriched at gene promoters and enhancers [43]. We therefore asked whether histone modifications were altered in *Pogz−/−* ESCs, by performing H3K27ac and H3K4me3 ChIP-seq experiments.

**Fig. 7:**
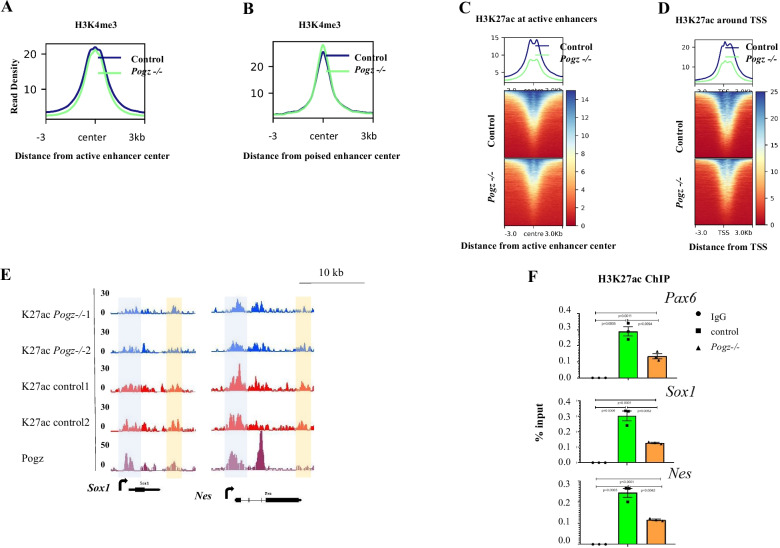
POGZ modulates enhancer activities. **A** Metaplot showing H3K4me3 ChIP-seq density at active enhancer regions in the presence and absence of POGZ. **B** Metaplot showing H3K4me3 ChIP-seq density at poised enhancer regions in the presence and absence of POGZ. **C** Up: Metaplot showing H3K27ac ChIP-seq density at active enhancer regions in the presence and absence of POGZ. Bottom: Heat map showing H3K27ac ChIP signals at active enhancers. **D** Up: Metaplot showing H3K27ac ChIP-seq density around TSS regions in the presence and absence of POGZ. Bottom: Heat map showing H3K27ac ChIP signals around TSS regions. **E** Snapshots showing H3K27ac ChIP-seq peaks at the indicated genes in control and *Pogz−/−* ESC-derived neurospheres. Orange: enhancers; Gray: promoters. **F** ChIP-PCR results showing H3K27ac enrichment at enhancer regions of the indicated neural genes in day 5 control and *Pogz−/−* ESC-derived neurospheres. ChIP were repeated two times

Global meta-analysis showed that H3K4me3 ChIP signals were barely altered in the absence of POGZ (Additional file 1: Figure S7A). When examining H3K4me3 at enhancer regions, we found that H3K4me3 levels were moderately reduced at active enhancers across a 6 kb window and were slightly elevated at poised enhancers near the peak midpoint (Fig. 7A–B). H3K4me3 signals around the proximal TSS regions were not changed (Additional file 1: Figure S7B). We identified 5970 H3K27ac peaks showing a more than twofold significant reduction in mutant ESCs, and 5556 H3K27ac peaks showing a more than twofold significant increase. We defined active enhancers in WT ESCs with H3K4me1/H3K27ac ChIP-seq and observed that there was a marked reduction of H3K27ac in mutant ESCs using a twofold change cutoff (Fig. 7C). We also observed a marked reduction of H3K27ac around the TSS proximal regions (Fig. 7D). Snapshots of representative neural genes such as *Pax6*, *Nes*, *Nkx2.9*, and *Nefm* and pluripotency genes such as *Nanog* and *Pou5f1* were shown (Additional file 1: Figure S7C). Enhancer activities positively correlate with H3K27ac levels [44, 45] and are important for gene activation. The above data suggested that POGZ primarily modulates enhancer activities, which is in line with that by analysis of POGZ-mediated chromatin accessibility.

We speculated that POGZ may also regulate enhancer activities during ESC neural induction, and that H3K27ac change contributes to the abnormal expression of neural progenitor genes during *Pogz−/−* ESC differentiation toward a neural fate. To investigate this, we performed H3K27ac ChIP-seq for day 5 control and *Pogz−/−* ESC-derived neurospheres. Although the global ChIP-seq signals of H3K27ac were barely altered in the absence of POGZ, reduced H3K27ac occupancy was observed at enhancer and promoter regions of neural progenitor genes including *Nes* and *Sox1* (Fig. 7E; Additional file 1: Figure S7D). We confirmed this by performing H3K27ac ChIP-PCR (Fig. 7F).

## Discussion

The roles of POGZ in ESCs and neurodevelopment remain unclear. Here, we show that POGZ plays important roles in the maintenance of ESC identity and ESC neural induction. POGZ functions as both a transcriptional activator and repressor by binding to gene promoter and enhancer regions in a DNA-sequence-specific manner. In addition, POGZ physically associates with the SWI-SNF (BAF) chromatin remodeler complex and recruits the esBAF to neural progenitor genes during ESC differentiation toward a neural fate. Loss of POGZ leads to change of chromatin accessibility and histone marks at local euchromatin loci, but not at a global level. Taken together, we propose that POGZ functions not only as a transcription factor, but also as a chromatin/epigenome regulator in ESCs and during ESC neural induction.

A salient feature of *Pogz−/−* and *Pogz+/− *ESCs is the apparent defects in cell proliferation and cell apoptosis. POGZ interacts with HP1 proteins who are known to play a key role in the maintenance of genome stability and in mitotic progression [1]. We speculated that prolonged loss of POGZ may lead to DNA damage or genome instability. Therefore, to investigate the direct effects of POGZ loss, we performed all the differentiation, ChIP-seq and ATAC-seq experiments using early passage *Pogz−/−* ESCs (mostly at passage 8–10 when cells remain ESC morphology and abundantly express OCT4 and NANOG).

In this work, the mutant ESC lines were generated by the CRISPR/Cas9 technology. We have validated the *Pogz* mutations by sequencing the top 5 predicted off-target genes. However, the genome integrity was not confirmed, in particular to the possible larger genomic deletion by CRISPR/Cas9 technology. It is possible that the phenotypes were driven by other genetic changes, for example due to chromothripsis.

*POGZ* is one of the top recurrently mutated genes in patients with NDDs, particularly ASD and ID. In addition to brain-related defects, *POGZ* patients may exhibit additional deficits, such as short stature, cardiac problem, hypotonia, strabismus, hearing loss, and abnormal craniofacial formation, including brachycephaly, long and flat malar region, broad nasal tip, short philtrum, and thin vermillion border [11]. However, the molecular and cellular mechanisms underlying the pleiotropic phenotypes by *POGZ* mutation remain unclear. Our work using ESC model leads to several important observations. First, POGZ is a master regulator of neural development. Both GO and KEGG analyses show that POGZ target genes are overwhelmingly linked with neural differentiation, neuronal and synapse function, leaning, and brain diseases. Consistently, neural progenitor genes failed to be up-regulated during *Pogz−/−* ESC EB formation as well as differentiation toward a neural fate, and the neural fibers are severely reduced in *Pogz−/−* ESC-derived neuronal cells. Second, POGZ physically associates the esBAF complex in ESCs and during ESC neural induction, and they together control chromatin accessibility and epigenome. As the esBAF complex is known to extensively regulate gene expression genome wide, it is not unexpected that germ layer- and signaling pathway-related genes are deregulated in the absence of POGZ. Third, histone modifications, such as H3K4me3 and H3K27ac, are altered in the absence of POGZ. It is well known that change of histone marks is closely related to abnormal gene expression in various cell types and brain disorders [43, 44].

POGZ has been shown to be associated with transcription repression and regulation of heterochromatin, as it is a reader of heterochromatin marks H3K9me2/3, interacts with heterochromatin protein HP1, and inhibits transcription by an in vitro luciferase assay [1–3]. In mouse brain tissues, POGZ forms a complex with HP1 and ADNP, members of a recently reported ChAHP repressive complex [15, 34]. In a previous LC–MS/MS assay, POGZ was identified as a candidate interactor for HP1 and CHD4. In this work, we surprisingly found that POGZ is closely linked with the esBAF chromatin remodeler complex. POGZ interacts with HP1; however, it fails to interact with CHD4 and ADNP in ESCs. We have examined the shared target sites among POGZ, ADNP, BRG1, and CHD4 in ESCs. POGZ and ADNP share 833 peaks, and POGZ and CHD4 share 1837 peaks; whereas this number is 6199 for POGZ and BRG1. This observation further supports that POGZ is more closely associated with esBAf/BRG1 than ADNP and CHD4. We propose that the major role of POGZ is involved in enhancer regulation at local euchromatin loci, likely modulating neural genes. However, POGZ is also co-localized with members of ChAHP complex and linked with heterochromatin marks such as H3K9me3/H4K20me3, suggesting that its role in these loci might be linked with gene repression [15]. The underlying mechanisms await further investigation.

We have performed ChIP-seq for H3K27ac and ATAC-seq for control and mutant ESCs, which allows us to compare the epigenetics with that by Markenscoff-Papadimitriou et al. We found that POGZ is predominantly localized to TSS and enhancer regions in ESCs, and globally, ATAC-seq signals were largely comparable between control and Pogz−/− ESCs. Further studies showed that loss of POGZ leads to reduced chromatin accessibility at enhancers, which was further supported by the reduced binding of H3K27ac, a mark for active enhancer. GO analysis of the genes associated with differentially accessible peaks revealed the enriched terms such as axonogenesis and regulation of neuron differentiation. In mouse brain tissues, Markenscoff-Papadimitriou et al. found that POGZ is predominantly localized to euchromatin regions, but not restricted to heterochromatin loci (with only 8% peaks in H4K20me3 marked heterochromatin). In the absence of POGZ, there is no overt genome-wide differences in chromatin accessibility and H3K27ac deposition; however, there was a decrease in chromatin accessibility and H3K27ac occupancy at enhancer regions of genes related to regulation of synapse function and axon development. Thus, both we and Markenscoff-Papadimitriou et al. have observed that at a global level, loss of POGZ had no significant impact on chromatin accessibility and H3K27ac occupancy, whereas it had significant roles at local loci where neural function-related genes are located. These observations are in line with that ESC differentiation toward neural fate is compromised and neural progenitor genes are down-regulated in the absence of POGZ.

Taken together, we propose that POGZ functions as both a transcription factor and a chromatin regulator. As a TF, POGZ can both activate and repress target genes by directly binding to the key regulatory elements. As an important chromatin/epigenomic regulator, POGZ can regulate chromatin by association with esBAF or CHD4 chromatin remodeler complexes depending on the cellular context. POGZ loss of function leads to abnormal gene expression and various developmental deficits, in particular the neural defects during neurogenesis.

## Limitations

This work is designed to study POGZ functions in ESCs and during ESC neural induction by generating *Pogz* homozygous mutants. The genotype and allele relevant to human neurodevelopmental disorders is heterozygous loss of function. To bring relevance to NDDs, more work should be performed using the heterozygous mutant ESCs.

## Conclusions

POGZ loss leads to compromised ESC identity and defects in neural induction and neurogenesis.

## Supplementary Information

**Additional file 1:** **Figure S1** Related to Figure 1. **A** Genotyping showing two additional mutant alleles: Mut2 and 3. **B** PH3 staining of control ESCs, Mut1- and Mut2-Pogz−/− ESCs at passage 12, showing reduced number of PH3 positive cells in homologous and heterozygous mutant ESCs. **C**Quantification of (B). **D** Apoptosis analysis by flow cytometry for control-, Mut1- (passage 12), Mut2- (passage 12), and FLAG-POGZ restoring ESCs. **E** The qRT-PCR results for the indicated genes of control, Mut1, and Mut2 ESCs at passage 18. **F** IF staining results showing that GATA6 was abnormally expressed in Mut2 and Mut3 Pogz−/− ESCs. Bar: 25 μm. **Figure S2** Related to Figure 2. **A** Morphology of day 1, 2, 6, and 8 EBs from control and two additional Pogz mutant ESCs (Mut2 and 3). **B** KEGG analysis of DEGs. **C**Morphology images during the formation of neurospheres and neuronal cells from control and Pogz−/− ESCs. Bar: 25 μm. **Figure S3** Related to Figure 3. **A** Double IF staining of HP1γ and FLAG-POGZ. Bar: 25 μm. **B** IP results showing that POGZ failed to pull down CHD4 in ESCs. **C** IP results showing that ADNP failed to pull down POGZ in ESCs. **D** IP results showing that POGZ failed to pull down ADNP in ESCs. **E** IP results showing FLAG-POGZ-Nter failed to pull down HA-BRG1-Nter in 293T cells. **F** IP results showing FLAG-POGZ-Cter failed to pull down HA-BRG1-Nter in 293T cells. **G** IP results showing FLAG-POGZ-Nter failed to pull down HA-BRG1-Cter in 293T cells. **H** IP results showing HA-BRG1-Cter failed to pull down FLAG-POGZ-Nter in 293T cells. **I** IP of in vitro synthesized POGZ and FLAG-BAF155 by TnT system. IP: POGZ, WB: FLAG. All experiments were repeated at least two times, and shown are representative images. **Figure S4** Related to Figure 4. **A** Snapshots of ChIP and CUT&Tag signals at Chchd1 and Dcp1a loci, showing that CUT&Tag outperforms FLAG ChIP-seq. Gray: proximal TSS; Orange: gene distal or gene body regions. **B** Snapshots ChIP and CUT&Tag peaks at the indicated loci, showing that POGZ is co-localized with H3K4me1 and H3K27ac. Gray: proximal TSS; Orange: enhancer regions. **C** A rank of motifs identified in POGZ binding sites by HOMER. Shown were the top 18 motifs with p value. **D** Snapshots of ChIP-seq and CUT&Tag peaks at Nanog and Pou5f1 loci, showing a broad overlapping occupancy of POGZ, OCT4, NANOG, H3K27Ac, ESRRB, KLF4, and MED1, indicating that POGZ is localized to super-enhancers. Orange: enhancer regions. **Figure S5** Related to Figure 5. **A**Genome-wide distribution patterns of POGZ and BRG1. **B** Snapshots showing the overlapping of POGZ, BRG1, BAF155, and SS18 at the indicated genes. **C** (Up) GO terms of BRG1+POGZ- genes; (Bottom) KEGG terms of BRG1+POGZ- genes. **D** (Up) GO terms of BRG1-POGZ+ genes; (Bottom) KEGG terms of BRG1-POGZ+ genes. **E** ChIP-PCR results showing BRG1 enrichment at promoters of the indicated genes in day 5 neurospheres from control and Pogz−/− ESCs. **Figure S6** **A** Heat map showing ATAC-seq signals in control and Pogz−/− ESCs. **B** Snapshots showing POGZ was enriched at ATAC-seq hypersensitive sites. **C** Top three motifs identified at POGZ-dependent ATAC-seq sites. **D** Metaplot showing that there was no change of chromatin accessibility at proximal TSS. **E** GO analysis of 148 genes that exhibited altered chromatin accessibility. **F** Snapshots showing that chromatin signals at proximal TSS and distal regions of the indicated genes. Gray: proximal TSS; Orange: distal regions. **G** Snapshots showing the ATAC-seq peaks at the indicated genes in the presence and absence of POGZ and BRG1. Gray highlighting the POGZ-bound DA peaks at the indicated loci. **Figure S7** Related to Figure 7. **A** Heat map showing H3K4me3 ChIP signals in control and Pogz−/− ESCs. **B**Heat map showing H3K4me3 signals around the proximal TSS in control and Pogz−/− ESCs. **C** Snapshots showing the ChIP-seq peaks of H3K4me3 and H3K27ac at the indicated genes in control and Pogz−/− ESCs. Neural and imprinted genes in black dashed box, and pluripotency genes in red dashed box. **D** Snapshots showing H3K27ac ChIP-seq peaks at the indicated genes in control and Pogz−/− ESC-derived neurospheres. Orange: enhancers; Gray: promoters

**Additional** **file 2.** GO terms of POGZ targets.

**Additional** **file 3.** KEGG terms of POGZ targets.

**Additional** **file 4.** Comparison of POGZ, ADNP, CBX3 inetractors in ESCS.

**Additional** **file 5.** Uncropped WB data images.

**Additional file 6**. POGZ, ADNP and CHD4 co-bound sites and genes.

**Additional** **file 7.** ATAC-seq and H3K27ac comparison between us and Markenscoff-Papadimitriou et al.

**Additional file 8.** POGZ targets and down-regulated genes in Figure 4F.

**Additional file 9.** POGZ targets and up-regulated genes in Figure 4G.

**Additional** **file 10**. Comparison of BRG1 and POGZ common target genes.

## Data Availability

All RNA-seq, ATAC-seq, ChIP-seq, and CUT&Tag data have been deposited in the public database at Beijing Genomic Institute (BGI) at https://bigd.big.ac.cn/, with the accession number of CRA003852. The *Pogz* mutant ESCs will be available to the community upon reasonable request.
