# Supplementary material for: Autism-associated protein POGZ controls ESCs and ESC neural induction by association with esBAF
Source: Mol Autism. 2022 Jun 1;13:24. doi: 10.1186/s13229-022-00502-9 (PMC9161502; doi:10.1186/s13229-022-00502-9)

Supplementary Figure 1

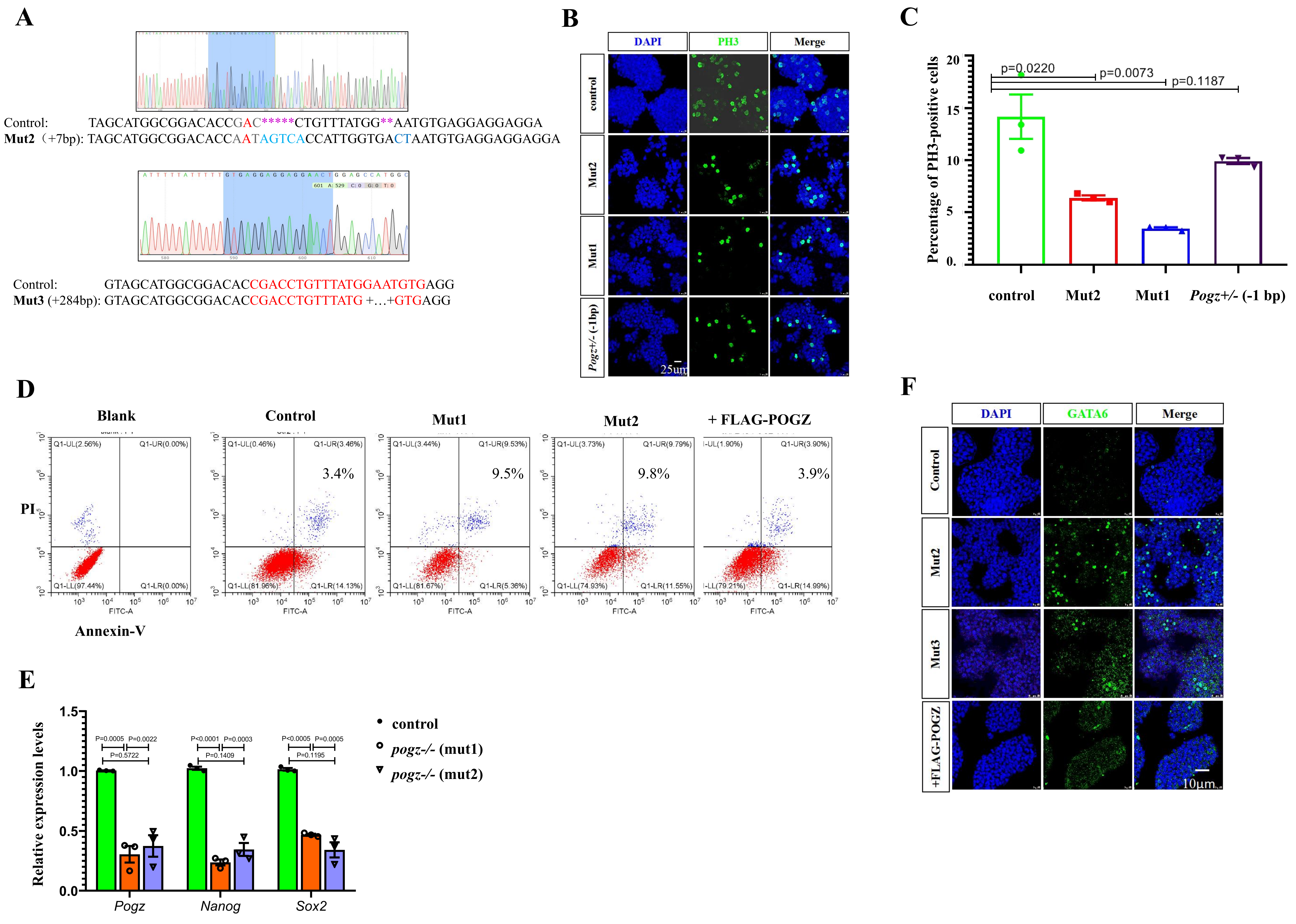

Supplementary Figure 2

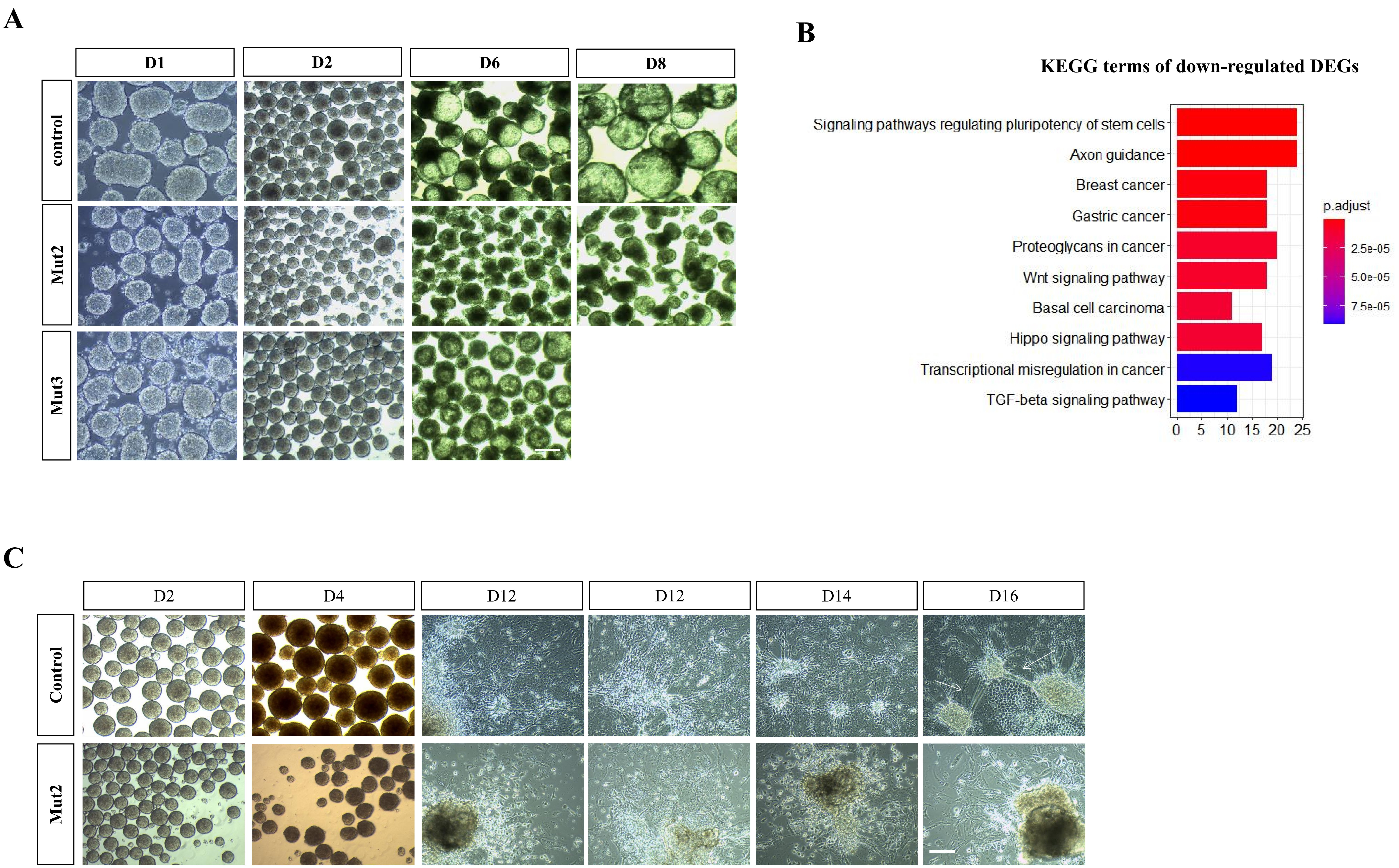

Supplementary Figure 3

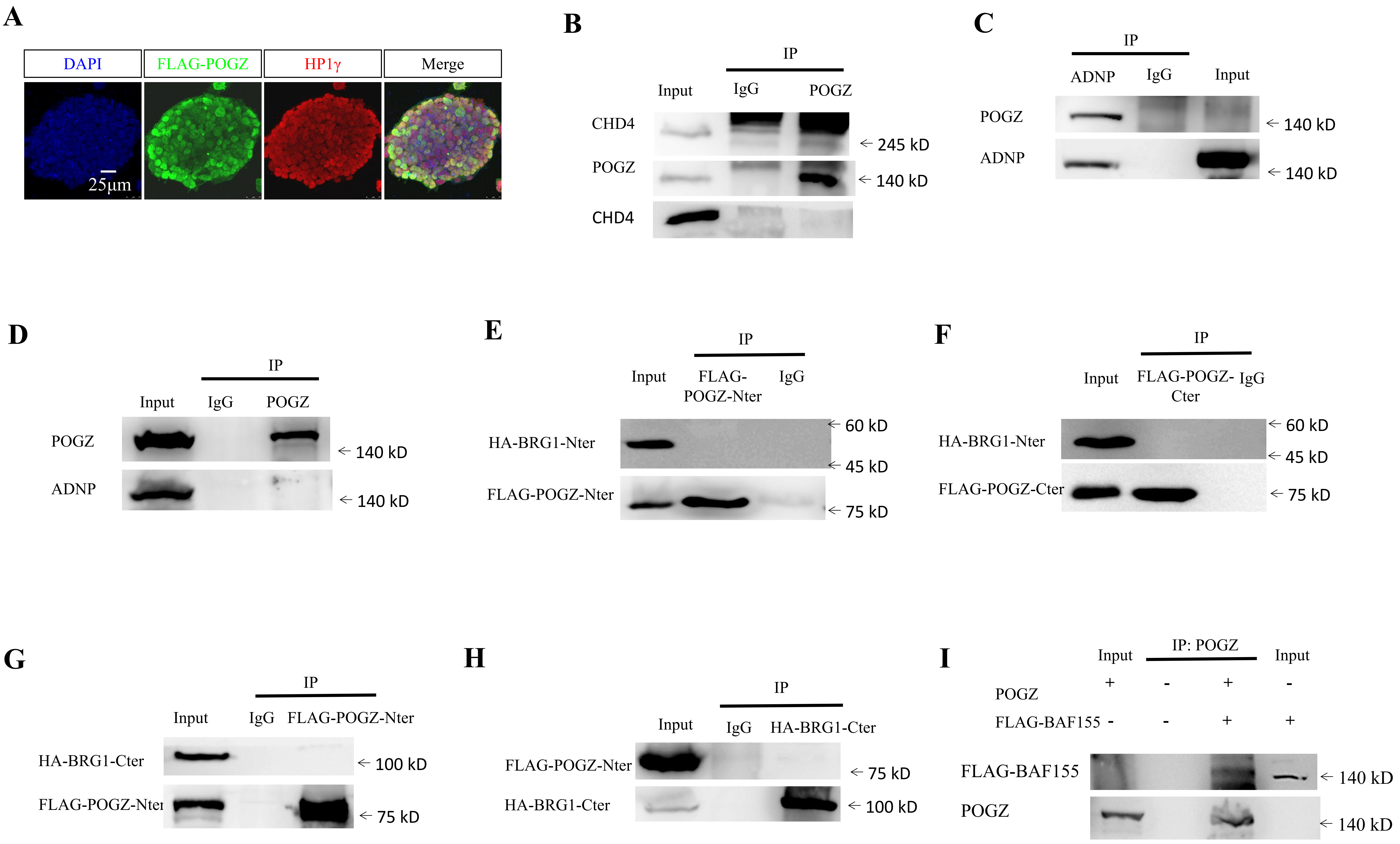

Supplementary Figure 4

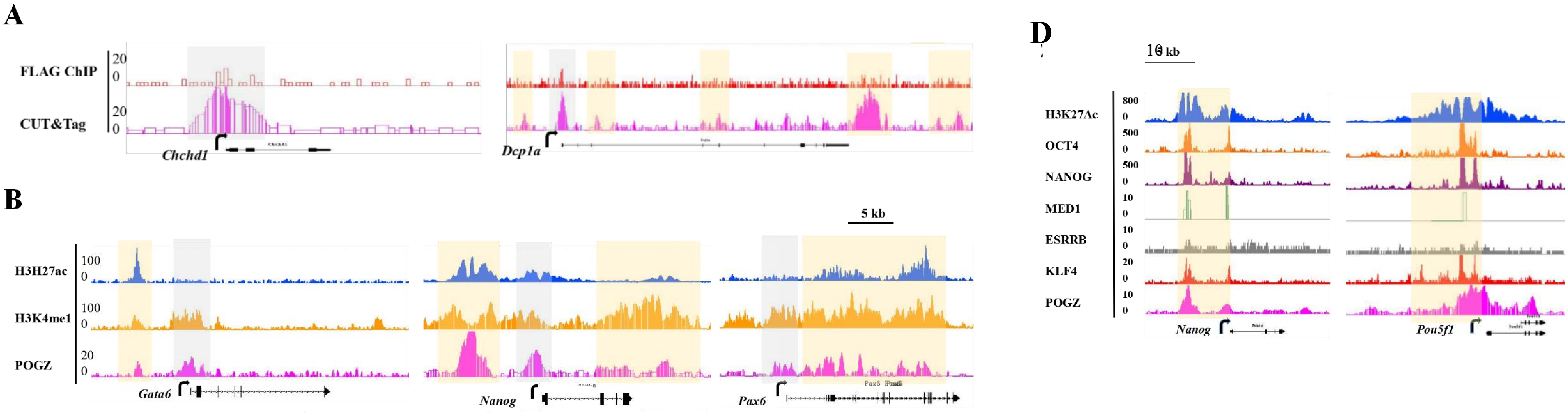

C

Rank	Motif	Name	P-value	log P-pvalue	q-value (Benjamini)	# Target Sequences with Motif	% of Targets Sequences with Motif	# Background Sequences with Motif	% of Background Sequences with Motif
1	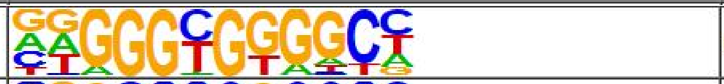	KLF1(Zf)/HUDEP2-KLF1-CutnRun(GSE136251)/Homer	1e-508	-1.170e+03	0.0000	4845.0	28.96%	4504.4	14.42%
2	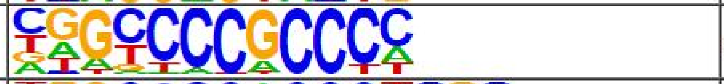	Sp2(Zf)/HEK293-Sp2.eGFP-ChIP-Seq(Encode)/Homer	1e-421	-9.706e+02	0.0000	6418.0	38.37%	7233.7	23.16%
3	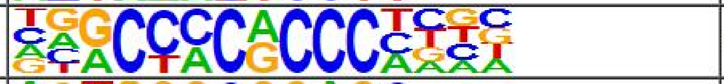	KLF3(Zf)/MEF-Klf3-ChIP-Seq(GSE44748)/Homer	1e-412	-9.506e+02	0.0000	3127.0	18.69%	2534.2	8.11%
4	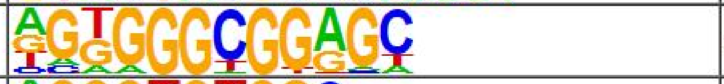	Sp5(Zf)/mES-Sp5.Flag-ChIP-Seq(GSE72989)/Homer	1e-407	-9.379e+02	0.0000	5089.0	30.42%	5261.8	16.85%
5	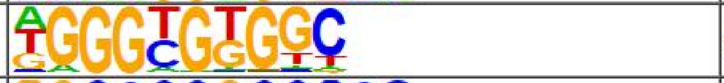	KLF5(Zf)/LoVo-KLF5-ChIP-Seq(GSE49402)/Homer	1e-368	-8.494e+02	0.0000	5599.0	33.47%	6216.1	19.90%
6	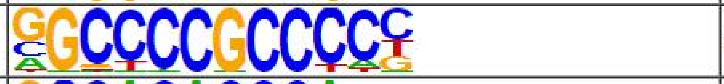	Sp1(Zf)/Promoter/Homer	1e-318	-7.336e+02	0.0000	2425.0	14.50%	1944.6	6.23%
7	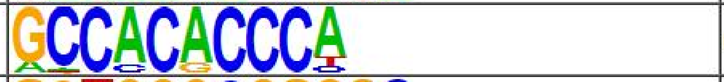	Klf4(Zf)/mES-Klf4-ChIP-Seq(GSE11431)/Homer	1e-271	-6.244e+02	0.0000	1976.0	11.81%	1537.7	4.92%
8	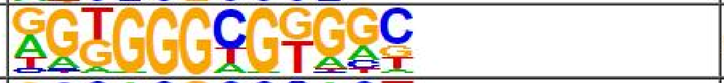	KLF14(Zf)/HEK293-KLF14.GFP-ChIP-Seq(GSE58341)/Homer	1e-249	-5.743e+02	0.0000	6685.0	39.96%	8693.9	27.83%
9	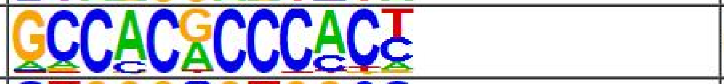	Klf9(Zf)/GBM-Klf9-ChIP-Seq(GSE62211)/Homer	1e-239	-5.505e+02	0.0000	2343.0	14.01%	2106.4	6.74%
10	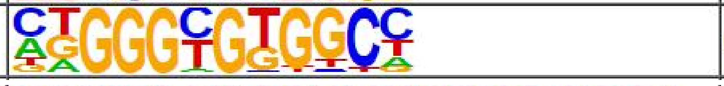	KLF6(Zf)/PDAC-KLF6-ChIP-Seq(GSE64557)/Homer	1e-238	-5.489e+02	0.0000	4523.0	27.04%	5260.6	16.84%
11	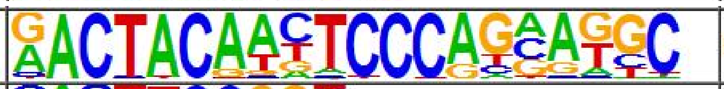	Ronin(THAP)/ES-Thap11-ChIP-Seq(GSE51522)/Homer	1e-149	-3.437e+02	0.0000	267.0	1.60%	58.5	0.19%
12	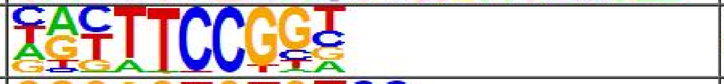	Elk4(ETS)/Hela-Elk4-ChIP-Seq(GSE31477)/Homer	1e-148	-3.425e+02	0.0000	2240.0	13.39%	2357.7	7.55%
13	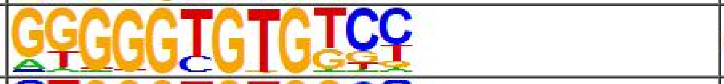	KLF10(Zf)/HEK293-KLF10.GFP-ChIP-Seq(GSE58341)/Homer	1e-134	-3.093e+02	0.0000	2261.0	13.52%	2464.8	7.89%
14	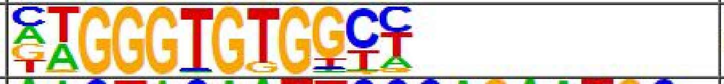	EKLF(Zf)/Erythrocyte-Klf1-ChIP-Seq(GSE20478)/Homer	1e-124	-2.869e+02	0.0000	820.0	4.90%	593.4	1.90%
15	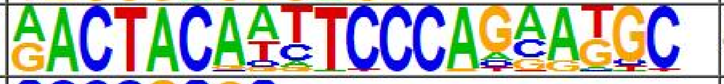	GFY-Staf(?,Zf)/Promoter/Homer	1e-115	-2.662e+02	0.0000	323.0	1.93%	121.5	0.39%
16	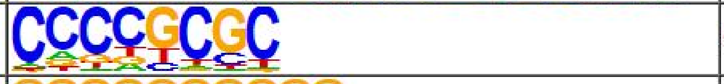	SUT1?/SacCer-Promoters/Homer	1e-114	-2.628e+02	0.0000	13319.0	79.62%	22478.2	71.97%
17	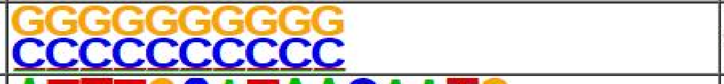	SeqBias: CG bias	1e-106	-2.456e+02	0.0000	15209.0	90.92%	26628.8	85.26%
18	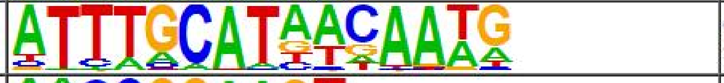	OCT4-SOX2-TCF-NANOG(POU,Homeobox,HMG)/mES-Oct4-ChIP-Seq(GSE11431)/Homer	1e-105	-2.424e+02	0.0000	505.0	3.02%	299.6	0.96%
19	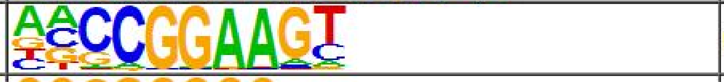	ELF1(ETS)/Jurkat-ELF1-ChIP-Seq(SRA014231)/Homer	1e-100	-2.309e+02	0.0000	1967.0	11.76%	2233.8	7.15%
20	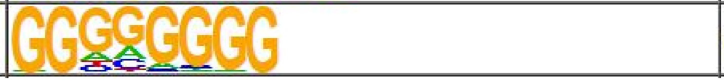	Maz(Zf)/HepG2-Maz-ChIP-Seq(GSE31477)/Homer	1e-100	-2.303e+02	0.0000	4972.0	29.72%	7062.5	22.61%

Supplementary Figure 5

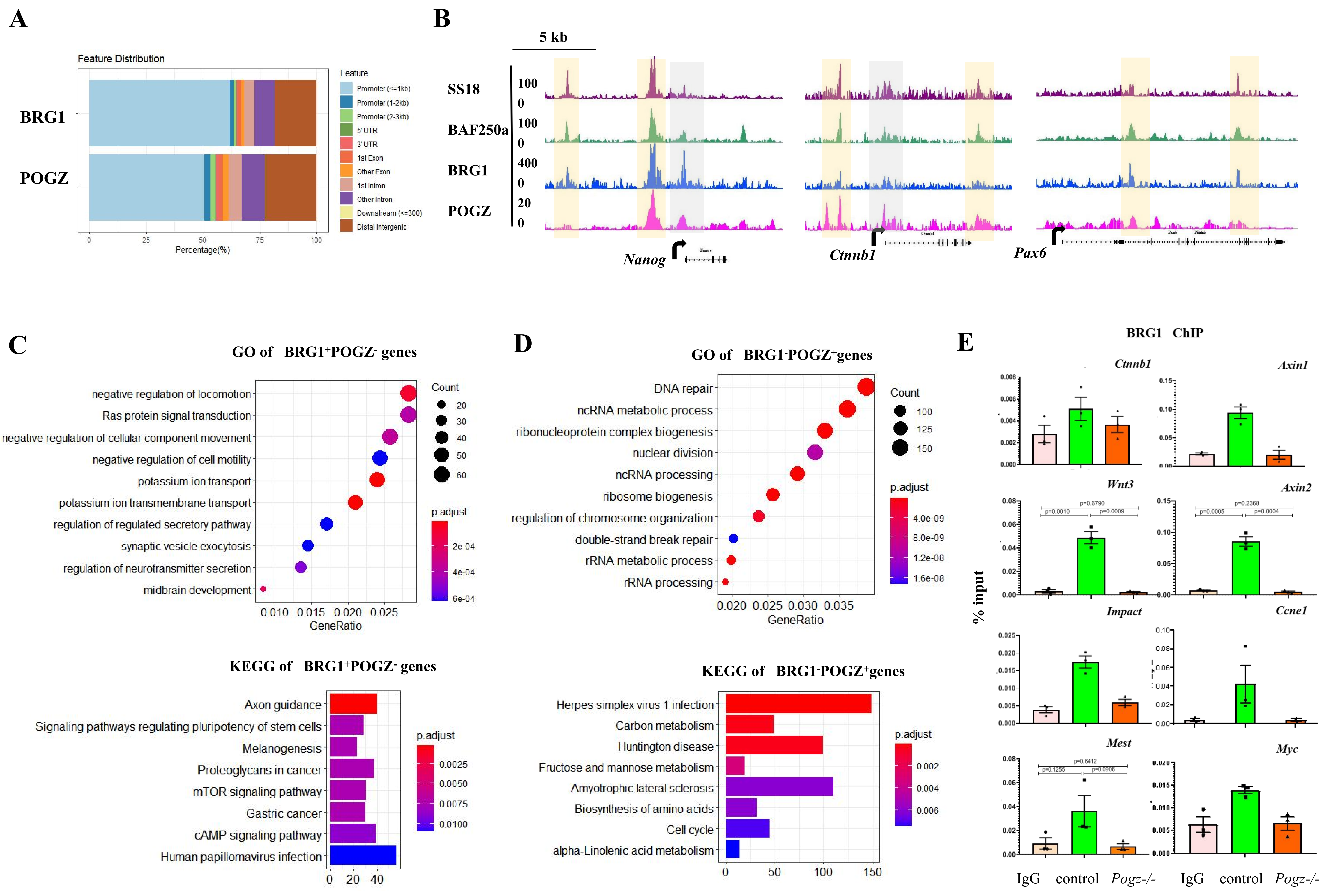

Supplementary Figure 6

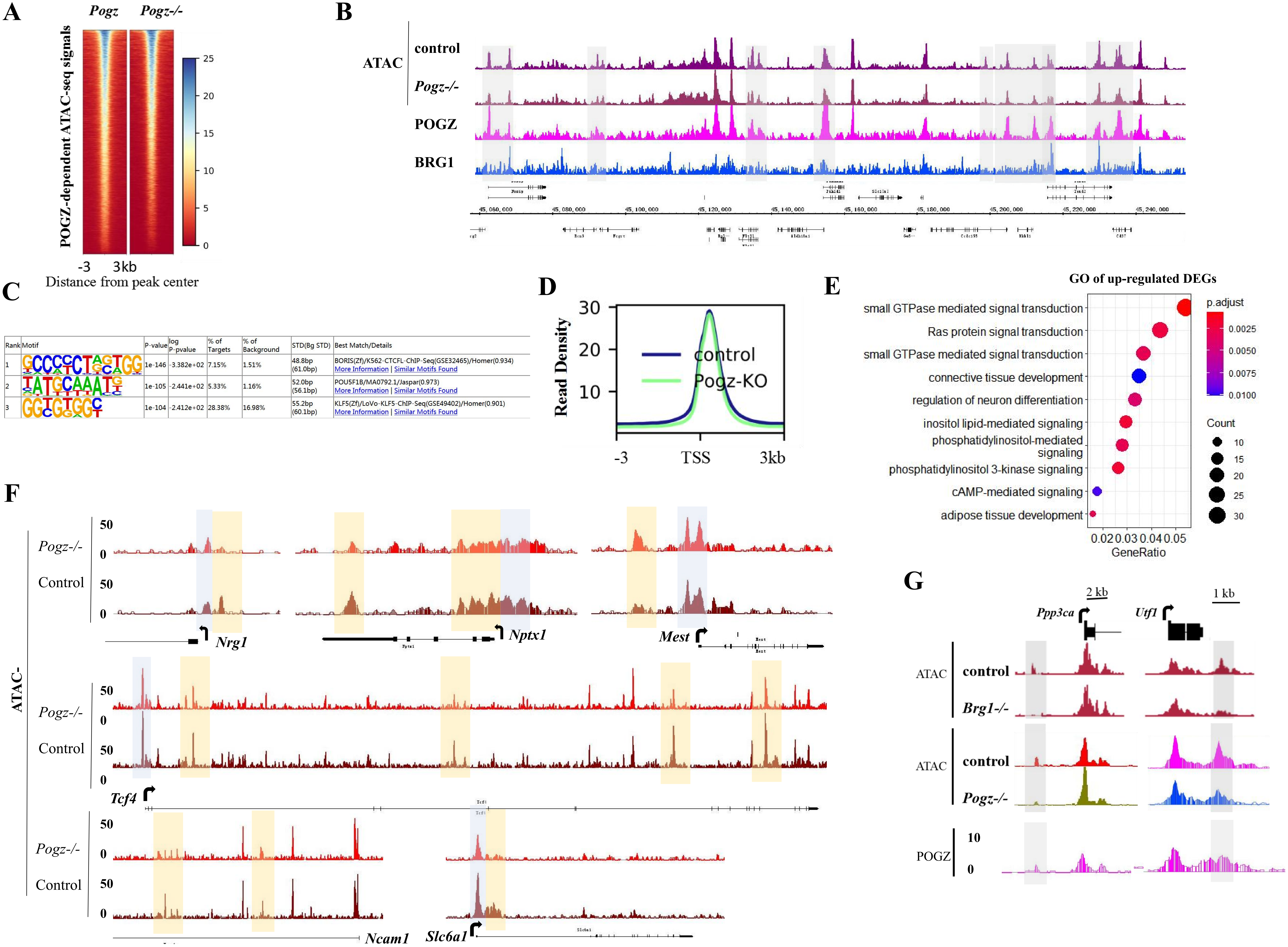

Rank	Motif	P-value	log P-pvalue	% of Targets	% of Background	STD(Bg STD)	Best Match/Details
1	GCCCCCTAGTGG	1e-146	-3.382e+02	7.15%	1.51%	48.8bp (61.0bp)	BORIS(Zf)/K562-CTCFL-ChIP-Seq(GSE32465)/Homer(0.934) More Information | Similar Motifs Found
2	TATGCAAATG	1e-105	-2.441e+02	5.33%	1.16%	52.0bp (56.1bp)	POU5F1B/MA0792.1/Jaspar(0.973) More Information | Similar Motifs Found
3	GGTGTGGC	1e-104	-2.412e+02	28.38%	16.98%	55.2bp (60.1bp)	KLF5(Zf)/LoVo-KLF5-ChIP-Seq(GSE49402)/Homer(0.901) More Information | Similar Motifs Found

Supplementary Figure 7

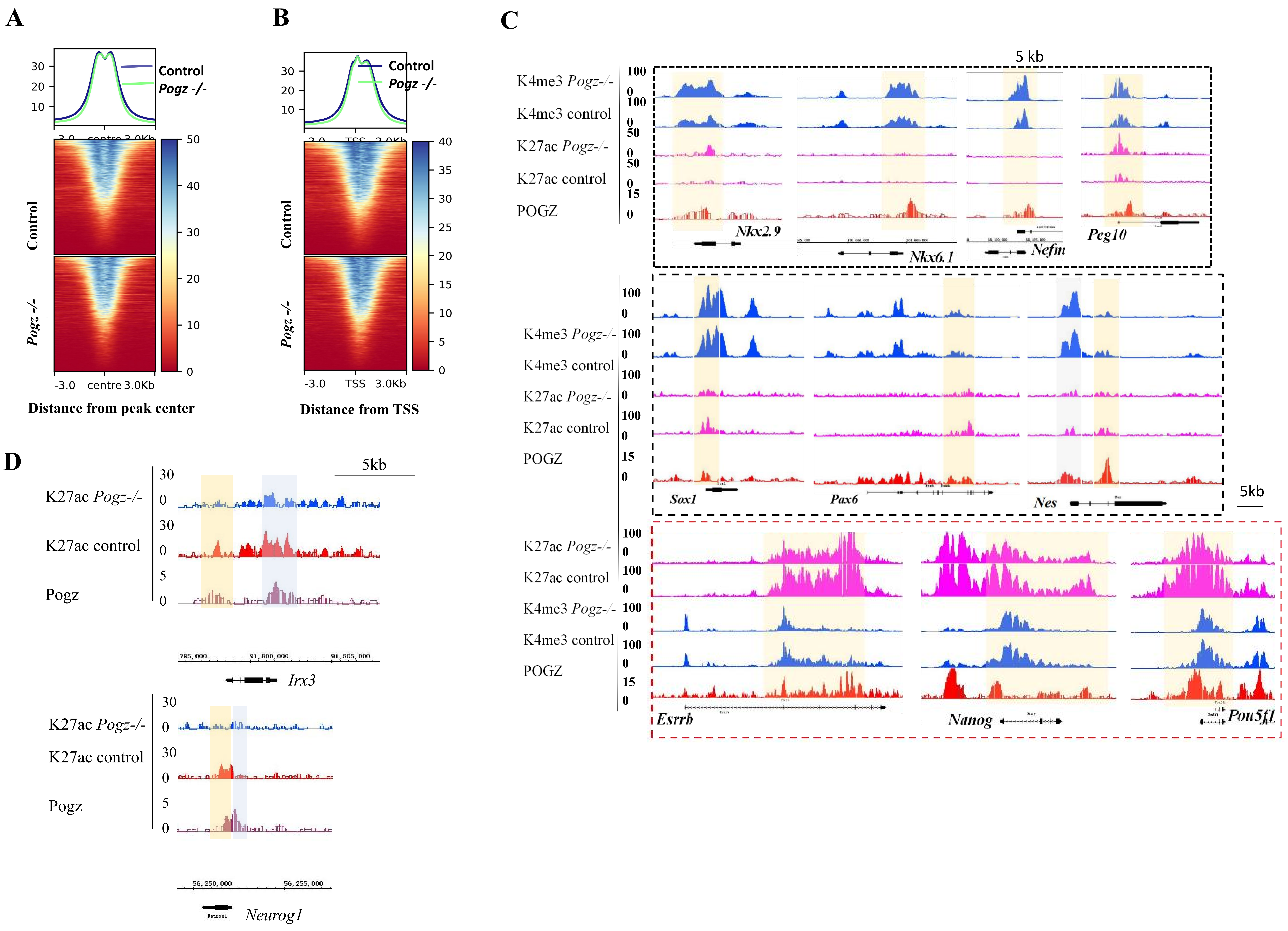

Supplement: Supplementary file 1 — Additional file 1: Figure S1 Related to Figure 1. A Genotyping showing two additional mutant alleles: Mut2 and 3. B PH3 staining of control ESCs, Mut1- and Mut2-Pogz−/− ESCs at passage 12, showing reduced number of PH3 positive cells in homologous and heterozygous mutant ESCs. CQuantification of (B). D Apoptosis analysis by flow cytometry for control-, Mut1- (passage 12), Mut2- (passage 12), and FLAG-POGZ restoring ESCs. E The qRT-PCR results for the indicated genes of control, Mut1, and Mut2 ESCs at passage 18. F IF staining results showing that GATA6 was abnormally expressed in Mut2 and Mut3 Pogz−/− ESCs. Bar: 25 μm. Figure S2 Related to Figure 2. A Morphology of day 1, 2, 6, and 8 EBs from control and two additional Pogz mutant ESCs (Mut2 and 3). B KEGG analysis of DEGs. CMorphology images during the formation of neurospheres and neuronal cells from control and Pogz−/− ESCs. Bar: 25 μm. Figure S3 Related to Figure 3. A Double IF staining of HP1γ and FLAG-POGZ. Bar: 25 μm. B IP results showing that POGZ failed to pull down CHD4 in ESCs. C IP results showing that ADNP failed to pull down POGZ in ESCs. D IP results showing that POGZ failed to pull down ADNP in ESCs. E IP results showing FLAG-POGZ-Nter failed to pull down HA-BRG1-Nter in 293T cells. F IP results showing FLAG-POGZ-Cter failed to pull down HA-BRG1-Nter in 293T cells. G IP results showing FLAG-POGZ-Nter failed to pull down HA-BRG1-Cter in 293T cells. H IP results showing HA-BRG1-Cter failed to pull down FLAG-POGZ-Nter in 293T cells. I IP of in vitro synthesized POGZ and FLAG-BAF155 by TnT system. IP: POGZ, WB: FLAG. All experiments were repeated at least two times, and shown are representative images. Figure S4 Related to Figure 4. A Snapshots of ChIP and CUT&Tag signals at Chchd1 and Dcp1a loci, showing that CUT&Tag outperforms FLAG ChIP-seq. Gray: proximal TSS; Orange: gene distal or gene body regions. B Snapshots ChIP and CUT&Tag peaks at the indicated loci, showing that POGZ is co-localized with H3K [file 13229_2022_502_MOESM1_ESM.pdf]
